# Hematopoietic stem cell transplantation ameliorates maternal diabetes–mediated gastrointestinal symptoms and autism‐like behavior in mouse offspring

**DOI:** 10.1111/nyas.14766

**Published:** 2022-02-27

**Authors:** Jiaying Zeng, Yujie Liang, Ruoyu Sun, Saijun Huang, Zichen Wang, Li Xiao, Jianpin Lu, Hong Yu, Paul Yao

**Affiliations:** ^1^ Department of Child HealthCare, Affiliated Foshan Maternity & Child Healthcare Hospital The Second School of Clinical Medicine of Southern Medical University Foshan P. R. China; ^2^ Department of Child Psychiatry, Kangning Hospital of Shenzhen Shenzhen Mental Health Center Shenzhen P. R. China; ^3^ Department of Pediatrics, Affiliated Foshan Maternity & Child Healthcare Hospital The Second School of Clinical Medicine of Southern Medical University Foshan P. R. China

**Keywords:** autism, gastrointestinal symptoms, hematopoietic stem cells, intestinal epithelial cells, maternal diabetes, autism spectrum disorder

## Abstract

Epidemiological studies have shown that maternal diabetes is associated with autism spectrum disorder development, although the detailed mechanism remains unclear. We have previously found that maternal diabetes induces persistent epigenetic changes and gene suppression in neurons, subsequently triggering autism‐like behavior (ALB). In this study, we investigated the potential role and effect of hematopoietic stem cells (HSCs) on maternal diabetes–mediated gastrointestinal (GI) dysfunction and ALB in a mouse model. We show *in vitro* that transient hyperglycemia induced persistent epigenetic changes and gene suppression of tight junction proteins. *In vivo*, maternal diabetes–mediated oxidative stress induced gene suppression and inflammation in both peripheral blood mononuclear cells and intestine epithelial cells, subsequently triggering GI dysfunction with increased intestinal permeability and altered microbiota compositions, as well as suppressed gene expression in neurons and subsequent ALB in offspring; HSC transplantation (HSCT) ameliorates this effect by systematically reversing maternal diabetes–mediated oxidative stress. We conclude that HSCT can ameliorate maternal diabetes–mediated GI symptoms and autism‐like behavior in mouse offspring.

## Graphical abstract

In this study, we investigated the potential role and effect of hematopoietic stem cells (HSCs) on maternal diabetes–mediated gastrointestinal (GI) dysfunction and ALB in a mouse model. Maternal diabetes induces persistent epigenetic changes and gene suppression in PBMCs, IECs, and neurons, while transplantation of HSCs ameliorates maternal diabetes–mediated GI dysfunction and ALB by systematically reversing maternal diabetes–mediated oxidative stress. We suggest that HSCs may play a potential therapeutic role in treatment of individuals with ASD.

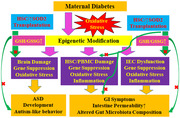

## Introduction

Autism spectrum disorders (ASDs) are a group of neurodevelopmental disorders primarily triggered by prenatal risk factor exposure and characterized by impaired social skills and restricted behaviors.^1^ The prevalence rate of ASD has increased to 1:54,^2^ with a male to female ratio of > 4:1.^3^ A variety of factors, including maternal diabetes^4^, ^5^, ^6^ and prenatal exposure to hormones (e.g., progestin and androgen),^7^, ^8^, ^9^, ^10^ have been reported to contribute to ASD development, while the detailed mechanism of their cause and related therapies remains largely unknown and unavailable, respectively.

Gastrointestinal (GI) symptoms have been reported to be associated with ASD,^11^ and the gut microbiota has been associated with brain dysfunction and ASD pathogenesis through the gut–brain axis. Furthermore, microbiota transfer therapy has been reported to ameliorate GI and ASD symptoms in autistic patients,^12^ while the cause–effect relationship between ASD and GI dysfunction (and related mechanisms) remains unclear.^13^, ^14^, ^15^, ^16^ Tight junction proteins, including claudin 1 (CLDN1), occludin (OCLN), and zonula occludens‐1 (ZO1), are the principal determinants of GI barrier function.^17^ Disruption of tight junctions is involved in GI symptoms in ASD patients, who show increased intestinal permeability;^18^, ^19^ increased diamine oxidase (DAO) activity^20^ and zonulin levels in serum^21^ are considered to be intestinal permeability markers in autistic subjects.

Hematopoietic stem cells (HSCs) located within the bone marrow are responsible for long‐term maintenance and generation of blood cell lineages.^22^, ^23^ We have previously reported that maternal diabetes induces epigenetic changes and gene suppression in neurons that, subsequently, contribute to autism‐like behaviors (ALBs) in offspring.^4^, ^5^, ^6^ Furthermore, maternal diabetes–mediated epigenetic modifications in HSCs can be inherited by peripheral blood mononuclear cells (PBMCs) during differentiation,^24^ subsequently inducing immune dysfunction in autism‐like offspring.^25^, ^26^ While HSC‐based therapeutic treatment has been shown to ameliorate ASD symptoms,^27^, ^28^ the underlying mechanism remains unclear and the strategy needs improvement.^29^

It has been early reported that transient hyperglycemia can induce persistent epigenetic changes and alter gene expression during subsequent normoglycemia.^30^ We have recently found that maternal diabetes induces persistent gene suppression of superoxide dismutase 2 (SOD2)^5^ and oxytocin receptor^4^ in brain regions, and these changes contribute to ALB in rodent offspring.^25^ Furthermore, in those studies, which used a maternal diabetes model of streptozotocin injection–induced type I diabetes, the subsequent mouse offspring showed ALBs in almost every individual, which included suppressed ultrasonic vocalization (USV), as well as impaired social recognition and interaction.^4^, ^6^, ^25^

In the present study, we evaluated the potential effect of maternal diabetes–mediated epigenetic changes and gene expression in the GI system of mouse offspring. We investigated the effects of HSC transfer on GI dysfunction and ALB with the use of a maternal diabetes–mediated autism‐like mouse model. *In vitro*, using human colon stem cells, we show that transient hyperglycemia induces persistent suppression of *CLDN1* and *ZO1* through epigenetic modifications, and that *SOD2* expression reverses, while short‐hairpin (sh)*SOD2* expression mimics, this effect. *In vivo*, we show that HSC transplantation (HSCT) ameliorates maternal diabetes–mediated oxidative stress and inflammation in PBMCs and intestinal epithelial cells (IECs), and it reverses tight junction protein suppression in IECs, GI dysfunction, and altered gut microbiota composition. We also show that HSCT ameliorates maternal diabetes–mediated ALB by reversing maternal diabetes–mediated systemic oxidative stress. We conclude that HSCT ameliorates maternal diabetes–mediated GI symptoms and ALB in autism‐like offspring. The results offer a possible therapeutic target, and potential mechanism of HSC‐mediated treatment, for individuals with ASD.

## Materials and methods

Detailed Materials and Methods section is described in Supplementary Materials (see File S1, online only), and primers for RT‐qPCR are provided in Table S1 (online only).

### Reagents and materials

The QualiCell® Human Colon Stem Cells‐XLC401 (#CSC‐C00314) and related maintenance media (#CM‐1339Z) were obtained from Creative Bioarray (Beijing, China). Antibodies specific for CLDN1 (sc‐166338), OCLN (sc‐133256), or ZO1 (sc‐33725) were obtained from Santa Cruz Biotechnology. Fluorescein isothiocyanate‐labeled dextran (FITC‐dextran, #46944) was obtained from Sigma.

### Mouse Sod2 cDNA

The cDNA for mouse *Sod2* was subcloned into the pLVX‐Puro vector (from Clontech) with the underlined restriction sites by following primers: *Sod2* forward primer: 5′‐ gtac‐ctcgag‐atg ttg tgt cgg gcg gcg tgc‐3′ (*Xho*1) and *Sod2* reverse primer: 5′‐ gtac‐tctaga‐tca ctt ctt gca agc tgt gta −3′ (*Xba*1).

### Preparation of lentiviruses

The lentivirus mouse plasmids for *shSod2* (sc‐41656‐SH) and nontarget control (sc‐108060) were obtained from Santa Cruz Biotechnology. The human *shSOD2*‐expressing lentivirus was prepared previously in our lab.^5^ Lentiviruses were expressed using Lenti‐X™ Lentiviral Expression Systems (from Clontech) according to the manufacturer's instructions.

### DNA methylation analysis

DNA methylation on the human *CLDN1* and *ZO1(TJP1*) promoters was determined by methylation‐specific PCR (MSP) analysis from previously established methods with minor modifications.^31^, ^32^ Briefly, genomic DNA was extracted and treated by bisulfite modification, and then was amplified using the following methylated and unmethylated primers. *CLDN1* methylated primer: forward 5′‐ ttt ata gga gcg aga aga ttt acg a −3′, reverse 5′‐ ccc taa cga ttt caa aac gac −3′; *CLDN1* unmethylated primer: forward 5′‐ ttt ata gga gtg aga aga ttt atg a −3′; reverse 5′‐ ccc cta aca att tca aaa caa c −3′. product size: 152 bp (methylated) & 153 bp (unmethylated); CpG island size: 172 bp; Tm: 66.0 °C. The final methylation readout was normalized by unmethylated input PCR. *ZO1* methylated primer: forward 5′‐ gtt ttt cgg aga tga aag tta tga c −3′, reverse 5′‐ taa aaa aac cga caa aac cga t −3′; *ZO1* unmethylated primer: forward 5′‐ agt ttt ttg gag atg aaa gtt atg at −3′; reverse 5′‐ taa aaa aac caa caa aac caa t −3′. product size: 152 bp (methylated) & 153 bp (unmethylated); CpG island size: 162 bp; Tm: 69.0 °C. The final methylation readout was normalized by unmethylated input PCR.

### *In vivo* mouse experiments

#### Protocol 1. Generation of chronic diabetic mice

Diabetic female mice were generated by injection of 35 mg/kg streptozocin (STZ) after 8‐h fasting. Blood glucose with > 250 mg/dL was considered diabetes positive. Females from either control (CTL) or STZ group were caged with males to generate subsequent offspring, and one offspring from each verified pregnant dam (*n* = 9 for each group) was used for the subsequent treatment in protocol 3.

#### Protocol 2. Preparation of hematopoietic stem cells with the manipulation of *Sod2* expression

Mouse HSCs were isolated and identified from 4‐ to 6‐week‐old male offspring donor mice from either CTL or STZ group, as described below.^33^, ^34^ The cells were then purified by density centrifugation using Histopaque 1083^®^ (#−1083‐1, Sigma) and resuspended in 10 mL of RPMI 1640 supplemented with 10% FBS and 2 mM EDTA. The isolated HSCs were infected by lentivirus for the expression of empty (EMP), *Sod2* expression (↑Sod2), or *Sod2* knockdown (shSod2) to develop HSC/EMP, HSC/↑Sod2, or HSC/shSod2 cells.

#### Protocol 3. Postnatal treatment by hematopoietic stem cell transplantation

Male offspring (6 weeks old) from either CTL or STZ group were used as recipients for HSCT. The recipient male mice were lethally irradiated with two doses of 6 Gy 3 h apart;^35^ and after 4 h of irradiation, 2 × 10^6^ HSCs prepared from protocol 2 were systemically transplanted by tail vein injection. The experimental mice were randomly separated into the following four groups. Group 1: offspring from CTL group received HSCT with HSCs from the CTL group infected with EMP lentivirus (CTL‐HSCT/CTL/EMP); group 2: offspring from STZ group received HSCT with HSCs from the STZ group infected with EMP lentivirus (STZ‐HSCT/STZ/EMP); group 3: offspring from STZ group received HSCT with HSCs from the STZ group infected with ↑Sod2 lentivirus (STZ‐HSCT/STZ/↑Sod2); and group 4: offspring from CTL group received HSCT with HSCs from the CTL group infected with shSod2 lentivirus (CTL‐HSCT/CTL/shSod2). All transplant recipient mice were set aside for a minimum of 12 weeks to allow for complete reconstitution of the bone marrow before they were used for evaluation of ALB and GI symptoms; brain tissues, including amygdala, hypothalamus, and hippocampus, were then collected. Whole blood was collected by heart puncture and the serum, and PBMCs were isolated. IECs were isolated as described below for further biological assays.

### Animal behavior test

The ALB test from mouse offspring was evaluated using USV, social interaction (SI) tests, and a three‐chambered social test as described in Supplementary Information (see Data S1, online only).

### Isolation and characterization of HSCs

The HSC preparation was a minor modification from previously described method.^33^ In brief, the whole bone marrow cells were collected from tibias in treated mice. Bone marrow cells were stained with antibodies for the identification of HSCs (c‐Kit^+^ Sca1^+^ Lin^–^), and the following antibodies were used: c‐Kit‐PE (#12‐1171‐82), Sca‐1‐FITC (#11‐5981‐82), and anti‐Lineage Antibody Cocktail comprises a mixture of PE‐Cy5‐conjugated antibodies, including anti‐B220, anti‐CD4, anti‐CD8, anti‐Gr‐1, anti‐Mac‐1, and anti‐TER119 (from eBioscience). For HSC sorting, debris, dead, and clumped cells were removed to obtain single and viable cells; then, the c‐Kit^+^ Sca1^+^ Lin^–^ cell population was isolated by HSC sorting; FACS analysis was performed on BD FACSMelody™ Cell Sorter.^34^

### Isolation of mouse IECs

The protocol for isolation of IECs was based on the previously described method with minor modifications. In brief, the small and large intestines were harvested individually from treated mice and rinsed extensively with RPMI‐1640 media (from Lonza) after Peyer's patches were removed (for small intestine). The rinsed intestines were opened longitudinally and macerated; the tissue pieces were shaken gently in RPMI‐1640 containing 2 mM EDTA and 10% fetal calf serum. The tissue preparations were passed through 70‐μm mesh filters, and the resulting single‐cell suspensions were applied to Percoll (from Sigma) density gradients of 25%, 40%, and 75%. After centrifugation at 2000× *g* for 20 min, the interface between the 25% and 40% layers was collected to obtain IECs. The cells were stained using antibodies for either epithelial cell adhesion molecule (EpCAM, from Biolegend) or CD45 (from Biolegend) and nucleic acid dye (Via‐Probe, from BD Biosciences). The Via‐Probe^–^ CD45^–^ EpCAM^+^ IECs were sorted using the BD FACSMelody^TM^ Cell Sorter (BD Biosciences) for further biological assays.^36^, ^37^

### Intestinal permeability assay

An intestinal permeability assay was followed based on the previously described method with minor modifications. In brief, treated mice were fasted for 4 h before the experiment and then, the FITC‐dextran (50 mg/mL, Cat# 46944 from Sigma) was gavaged (600 mg/kg). After 4 h, whole blood was collected by cardiac puncture and placed at room temperature for 1 h before being centrifuged at a speed of 3000 rpm for 10 minutes. The supernatant was then transferred to a new tube for further centrifugation at a speed of 12,000 rpm for 10 min at 4 °C. The subsequent supernatant (serum) was diluted with equal volume of PBS, and then 100 μL of the diluted serum was added to a 96‐cell microplate. The concentration of FITC in the serum was determined at excitation/emission wavelengths of 485/530 nm using an FL×800 microplate fluorescence reader (Bio‐Tek). Serial diluted FITC‐dextran (0, 0.5, 1, 2, 4, 6, 8, and 10 μg/μL) was used as standards. Serum of mice administered PBS was used as a negative control.^14^, ^38^

### Fecal microbiome analysis

Fecal samples of the experimental mice were collected and stored at −80 °C before being processed. Microbial DNA was extracted using a QIAamp Fast DNA Stool Mini Kit (from Qiagen) according to the manufacturer's protocol.^39^ The purity and concentration of the extracted DNA were evaluated using agarose gel electrophoresis. The fecal microbiota was studied by performing V3–V4 16S rDNA amplicon sequencing to obtain operational taxonomic units (OTU) defining the bacterial communities.^40^ Sequencing samples from frozen fecal pellets were prepared, sequenced, and subsequently processed using the MiSeq Pe300 Sequencing Platform (from Illumina) by Shanghai OE Biotech Inc. The raw data were treated and processed using a QIIME 2^TM^ software package, and the subsequent sequences of OTU were blasted in the Silva database (version 138). The alpha and beta diversities were analyzed using QIIME 2^TM^ software package.^14^

### Serum biochemical analysis

The whole blood was collected from experimental mice by heart puncture and the serum was prepared by centrifugation at 2000× *g* for 15 minutes. The specimens were then stored at −80 °C until analysis. The GSH/GSSG ratio was determined using a GSH/GSSG Ratio Detection Assay Kit (Fluorometric‐Green) (#ab138881) from Abcam, while DAO activity was measured by DAO ELISA Kit (#MBS160374) and the zonulin levels were determined by Mouse Zonulin ELISA Kit (#NC1314884) from MyBioSource according to the manufacturers’ instructions.^21^, ^41^

### Analysis of cytokines

Mouse cytokines from either the serum or cell supernatant, IL‐1β, IL‐6, IL‐17a, and MCP1, were measured using the Bio‐Plex Pro Mouse Cytokine 23‐plex Assay kit (#M60009RDPD from BioRad) and Bio‐Plex 200 Systems (BioRad) according to the manufacturer's instructions. Protein concentration in lysates was determined using the Coomassie Protein Assay Kit (Pierce Biotechnology) according to the manufacturer's instructions; lysates were adjusted to 200 mg/mL with extraction buffer; 50 mL lysates were diluted two‐fold in sample dilution buffer and analyzed in duplicates. Analytes were quantified in each sample against a calibration curve of known concentrations.^42^

## Results

### Transient hyperglycemia triggers persistent gene suppression during subsequent normoglycemia through consistent oxidative stress

**Figure 1:**
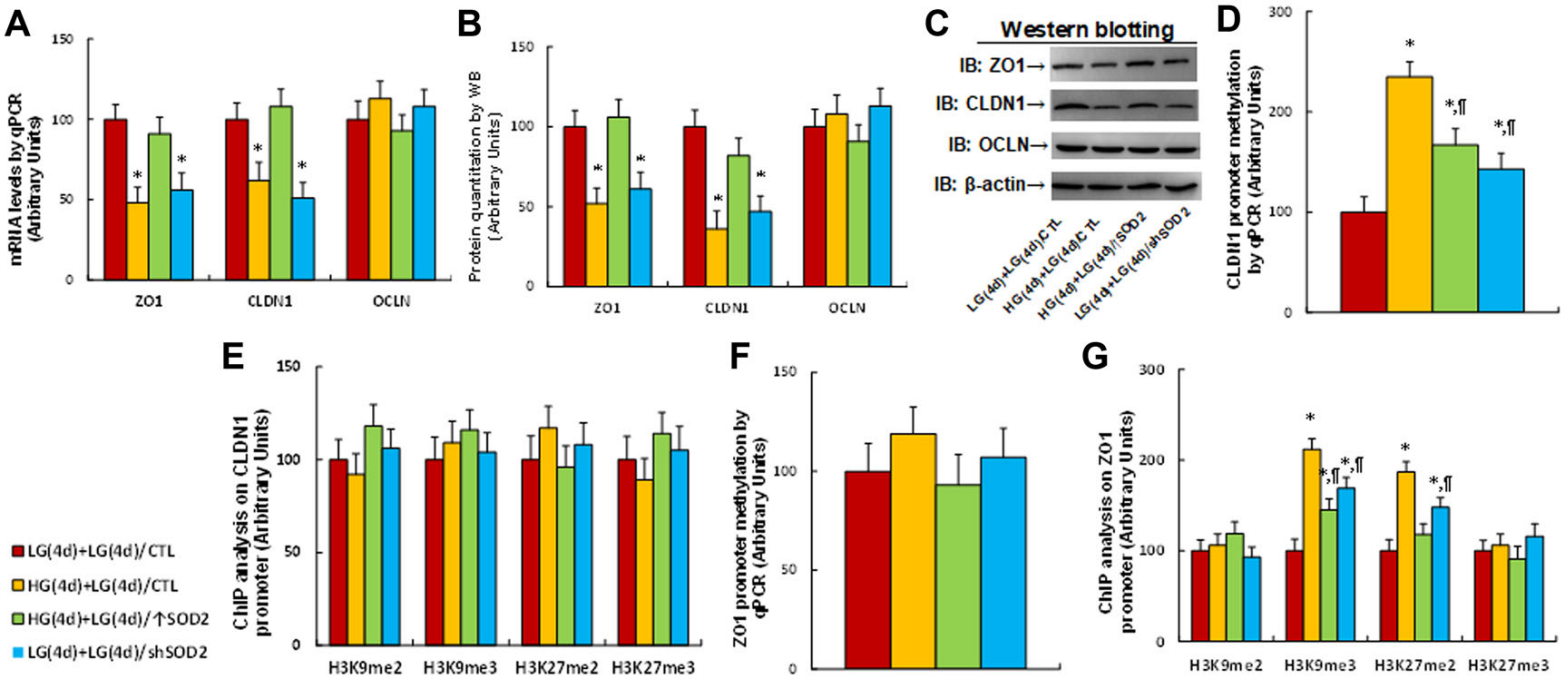
Transient hyperglycemia triggers persistent gene suppression during subsequent normoglycemia through consistent oxidative stress. Human colon stem cells were treated with either 5 mM low glucose (LG) or 25 mM high glucose (HG) for 4 days. The cells were then infected by control (CTL), *SOD2* overexpression (↑SOD2), or *SOD2* mRNA knockdown (shSOD2) lentivirus for 1 day before they were treated by LG for another 4 days in the presence of 1% serum; the cells were then harvested for further analysis. (A) mRNA levels, *n* = 4. (B) Quantitation of protein levels, *n* = 5. (C) Representative immunoblots for B. (D) DNA methylation on the *CLDN1* promoter, *n* = 4. (E) ChIP analysis on the *CLDN1* promoter, *n* = 4. (F) DNA methylation on the *ZO1* promoter, *n* = 4. (G) ChIP analysis on the *ZO1* promoter, *n* = 4. ^*^ *P* < 0.05 versus LG(4d) + LG(4d)/CTL group; ^¶^ *P* < 0.05 versus HG(4d) + LG(4d)/CTL group. Data are expressed as mean ± SD.

We evaluated the potential effect of transient hyperglycemia on the expression of tight junction proteins ZO1, CLDN1, and OCLN. Human colon stem cells were treated with either 5 mM low glucose (LG) or 25 mM high glucose (HG) for 4 days (4d) and then infected by lentiviruses control (CTL), *SOD2* overexpression (↑SOD2), or *SOD2* knockdown (shSOD2) for 1 day before they were further treated by LG for another 4 days. The results showed that 4‐day hyperglycemia plus 4‐day normoglycemia (HG(4d) + LG(4d)/CTL) treatment significantly suppressed mRNA levels of *ZO1* and *CLDN1*, compared with the LG plus 4‐day normoglycemia (LG(4d) + LG(4d)/CTL) treatment. Cells with *SOD2* overexpression under hyperglycemia (HG(4d) + LG(4d)/↑SOD2) did not show suppressed mRNA levels of *ZO1* and *CLDN1*, while knockdown of *SOD2* under LG (LG(4d) + LG(4d)/shSOD2) mimicked the HG effect. In contrast to *ZO1* and *CLDN1*, *OCLN* mRNA did not differ significantly among the treatment groups (Fig. 1A). We also measured protein levels, and an expression pattern consistent with the mRNA levels was observed (Fig. 1B and C; and Fig. S1A, online only).

We then evaluated the possible effect of epigenetic modifications on the *ZO1* and *CLDN1* promoters. The results showed that HG(4d) + LG(4d)/CTL treatment significantly increased DNA methylation on the *CLDN1* promoter (Fig. 1D) but had no effect on H3K9 and H3K27 modifications (Fig. 1E). Treatment did not change DNA methylation on the *ZO1* promoter (Fig. 1F), but significantly increased H3K9me3 and H3K27me2 modifications (Fig. 1G), compared with LG (LG(4d) + LG(4d)/CTL) treatment. *SOD2* overexpression (HG(4d) + LG(4d)/↑SOD2) partly reversed, while *SOD2* knockdown under LG (LG(4d) + LG(4d)/shSOD2) partly mimicked, this effect. Furthermore, we evaluated the possible effect of hyperglycemia and *SOD2* expression on H4 methylation and histone acetylation on the *CLDN1* (Fig. S2, online only) and *ZO1* (Fig. S3, online only) promoters; neither showed any changes. We concluded that transient hyperglycemia triggers epigenetic changes and gene suppression during subsequent normoglycemia.

### Transplantation of *Sod2*‐expressing HSCs ameliorates, while *shSOD2*‐expressing HSCs mimic, maternal diabetes–mediated oxidative stress in PBMCs

**Figure 2:**
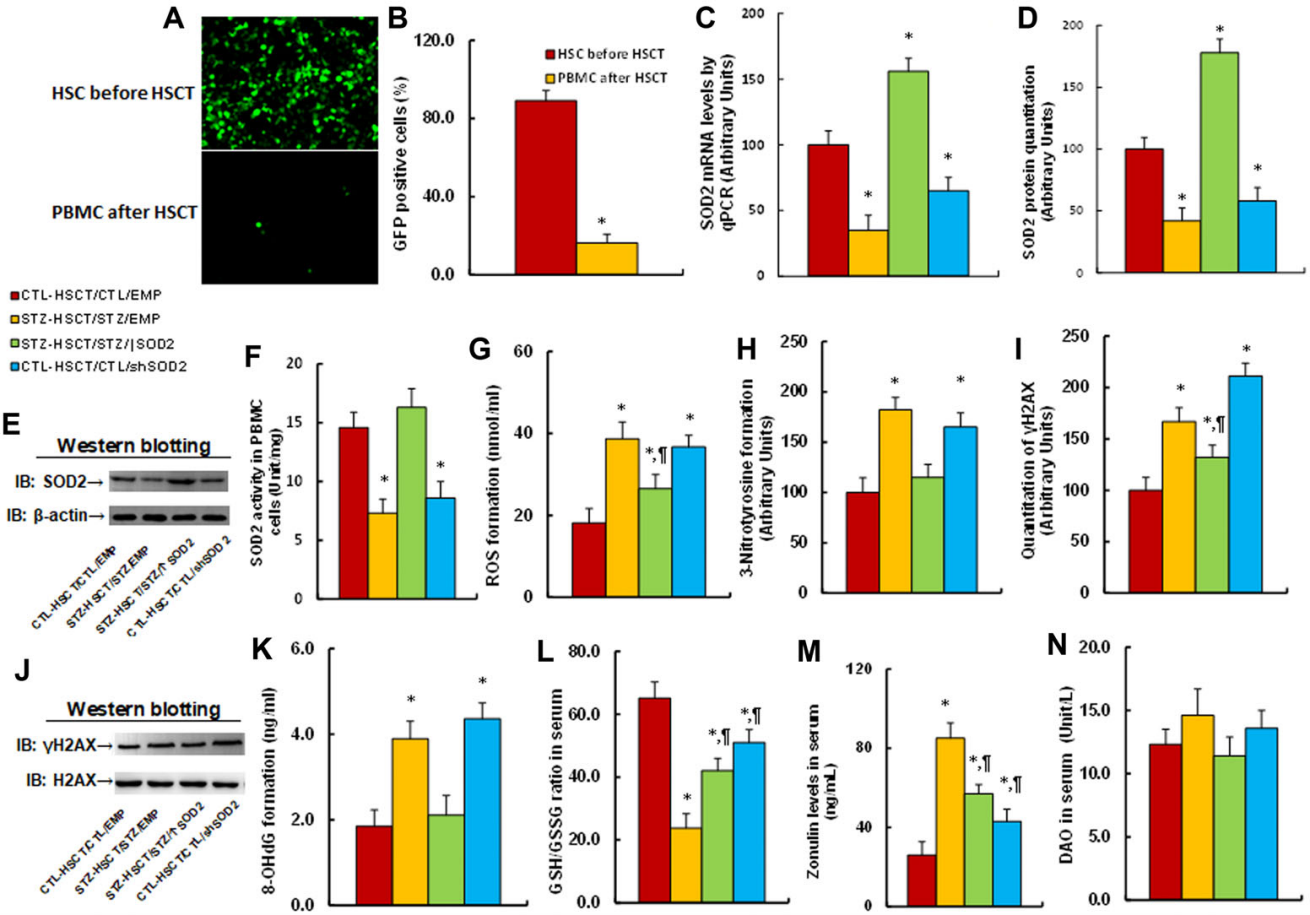
Transplantation of *Sod2*‐expressing HSCs ameliorates, while transplantation of *shSod2*‐expressing HSCs mimics, maternal diabetes–mediated oxidative stress in PBMCs. Male offspring from either CTL or STZ dams received HSCT with HSCs infected by either *Sod2*‐ or *shSod2*‐expressing lentivirus for biomedical analysis. GFP lentivirus–infected HSCs were transplanted; the PBMCs from recipient mice were collected and the GFP^+^ cells were counted: (A) representative GFP pictures for two different treatments, including HSCs before HSCT and PBMCs after HSCT; (B) quantitation of GFP^+^ cells for A, *n* = 5. ^*^ *P* < 0.05 versus HSCs before HSCT group. PBMCs were collected from recipient mice after HSCT for biomedical analysis: (C) *SOD2* mRNA levels, *n* = 4; (D) quantitation of SOD2 protein levels, *n* = 5; (E) representative immunoblots for D; and (F) SOD2 activity, *n* = 5. (G) ROS formation, *n* = 5. (H) 3‐nitrotyrosine formation, *n* = 5. (I) Quantitation of γH2AX formation, *n* = 5. (J) Representative immunoblots for I. (K) 8‐OHdG formation, *n* = 5. Serum was collected from recipient mice after HSCT for biomedical analysis: (L) GSH/GSSG ratio, *n* = 55; (M) Zenulin levels, *n* = 5; and (N) DAO activity, *n* = 5. ^*^ *P* < 0.05 versus CTL‐HSCT/CTL/EMP group; ^¶^ *P* < 0.05 versus STZ‐HSCT/STZ/EMP group. Data are expressed as mean ± SD.

We evaluated the potential effect of HSCT and *Sod2* expression on maternal diabetes–mediated oxidative stress in a mouse model. Male offspring from either CTL or STZ dams received HSCT with HSCs that were infected by either *Sod2*‐ or *shSod2*‐expressing lentivirus. We first determined whether transplanted HSCs could differentiate into PBMCs after HSCT. GFP lentivirus infected–HSCs were transplanted; the PBMCs from recipient mice were collected 12 weeks later and GFP^+^ cells were counted. The data showed that before HSCT, 89.1% of HSCs were infected by GFP‐expressing lentivirus, while after HSCT, 16.3% of PBMCs were GFP^+^ cells (Fig. 2A and B), indicating that HSCs can differentiate into PBMCs after HSCT.

We then evaluated the potential effect of HSCT on endogenous *Sod2* expression in PBMCs in the context of induced maternal diabetes, including effects on *Sod2* mRNA (Fig. 2C), SOD protein level (Fig. 2D and E; and Fig. S1B, online only), and SOD2 activity (Fig. 2F). The data showed that maternal diabetes induction (STZ‐HSCT/STZ/EMP) significantly decreased endogenous *Sod2* expression, compared with the control (CTL‐HSCT/CTL/EMP) group. STZ‐HSCT/STZ/↑Sod2 treatment reversed, while CTL‐HSCT/CTL/shSod2 treatment mimicked, this effect.

We also evaluated the potential effect on oxidative stress in PBMCs, including the formation of reactive oxygen species (ROS) (Fig. 2G), 3‐nitrotyrosine (Fig. 2H), γH2AX (Fig. 2I and J; Fig. S1C, online only), and 8‐OHdG (Fig. 2K). The data indicated that maternal diabetes induction (STZ‐HSCT/STZ/EMP) significantly increased oxidative stress, compared with the control (CTL‐HSCT/CTL/EMP) group; STZ‐HSCT/STZ/↑Sod2 treatment partly reversed, while CTL‐HSCT/CTL/shSod2 treatment partly mimicked, this effect.

Finally, we evaluated the effect of HSCT on autism markers in serum; we found that maternal diabetes induction (STZ‐HSCT/STZ/EMP) significantly decreased the ratio of GSH to GSG (Fig. 2I) and increased zenulin levels (Fig. 2M) but had no effect on DAO activity (Fig. 2N), compared with the control (CTL‐HSCT/CTL/EMP) group. STZ‐HSCT/STZ/↑Sod2 treatment partly reversed, while CTL‐HSCT/CTL/shSod2 treatment partly mimicked, this effect.

Together, the above results indicated that HSCT with increased *Sod2* expression ameliorates maternal diabetes–mediated oxidative stress in PBMCs.

### Transplantation of *Sod2*‐expressing HSCs ameliorates, while *shSod2*‐expressing HSCs mimic, maternal diabetes–mediated inflammation in PBMCs

**Figure 3:**
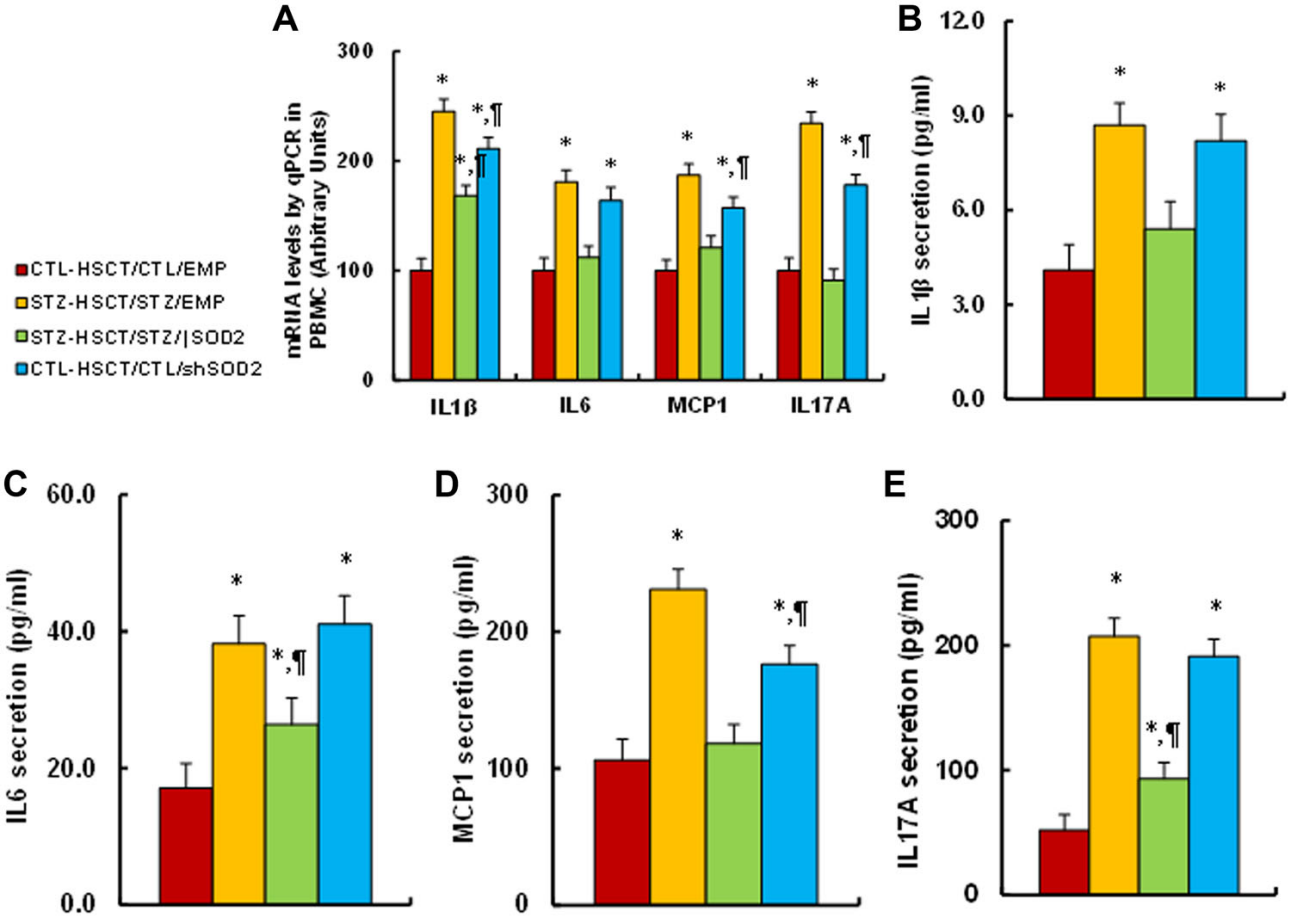
Transplantation of *Sod2*‐expressed HSCs ameliorates, while transplantation of *shSod2*‐expressing HSCs mimics, maternal diabetes–mediated inflammation in PBMC. Male offspring from either CTL or STZ dams received HSCT with HSCs that were infected by either Sod2‐ or shSod2‐expressing lentivirus, and the PBMCs were isolated for analysis of proinflammatory cytokine release. (A) mRNA levels of preinflammatory cytokines, *n* = 4. (B) IL‐1β secretion, *n* = 9. (C) IL‐6 secretion, *n* = 9. (d) MCP1 secretion, *n* = 9. (E) IL17A secretion, *n* = 9. ^*^ *P* < 0.05 versus CTL‐HSCT/CTL/EMP group; ^¶^ *P* < 0.05 versus STZ‐HSCT/STZ/EMP group. Data are expressed as mean ± SD.

Next, we evaluated the potential effect of HSCT and *SOD2* expression on maternal diabetes–mediated inflammation in PBMCs. We first evaluated mRNA levels of proinflammatory cytokines IL‐1β, IL‐6, MCP1, and IL‐17A. The data showed that maternal diabetes induction (STZ‐HSCT/STZ/EMP) significantly increased mRNA levels of the cytokines, compared with the control (CTL‐HSCT/CTL/EMP) group. STZ‐HSCT/STZ/↑Sod2 treatment partly reversed, while CTL‐HSCT/CTL/shSod2 treatment partly mimicked, this effect (Fig. 3A). We also evaluated IL‐1β (Fig. 3B), IL‐6 (Fig. 3C), MCP1 (Fig. 3D), and IL‐17A (Fig. 3E) secretion from PBMCs and found a pattern consistent with that of the cytokine mRNA levels, indicating that HSCT with increased *SOD2* expression ameliorates maternal diabetes–mediated inflammation in PBMCs.

### Potential effect of HSCT with increased *Sod2* expression on maternal diabetes–mediated gene regulation in IECs

**Figure 4:**
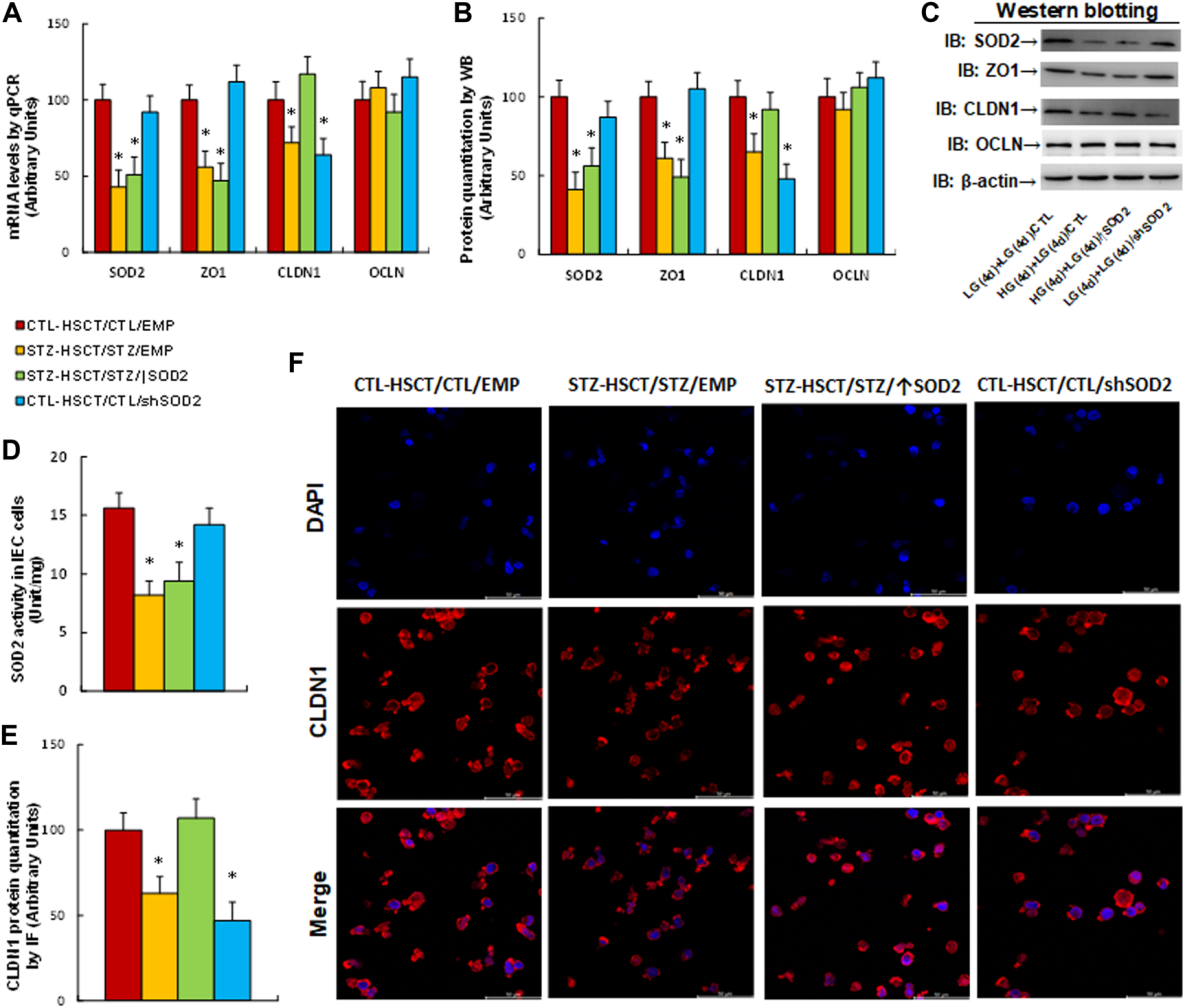
Potential effect of HSCT with increased *Sod2* expression on maternal diabetes–mediated gene regulation in IECs. Male offspring from either CTL or STZ dams received HSCT with HSCs infected with either *Sod2*‐ or *shSod2*‐expressing lentivirus, and the IECs were isolated for biomedical analysis. (A) mRNA levels by qPCR, *n* = 4. (B) Quantitation of protein levels, *n* = 5. (C) Representative immunoblots for B. (D) SOD2 activity, *n* = 5. (E) Quantitation of CLDN1 protein, *n* = 5. (F) Representative immunoblots for E. ^*^ *P* < 0.05, CTL‐HSCT/CTL/EMP group. Data are expressed as mean ± SD.

Next, we evaluated the potential effect on gene expression of *Sod2* and the tight junction protein genes *Zo1*, *Cldn1*, and *Ocln*. We found that maternal diabetes induction (STZ‐HSCT/STZ/EMP) significantly decreased mRNA levels of *Sod2*, *Zo1*, and *Cldn1*, compared with the control (CTL‐HSCT/CTL/EMP) group. STZ‐HSCT/STZ/↑Sod2 treatment reversed, while CTL‐HSCT/CTL/shSod2 treatment mimicked, the effect on *Cldn1* mRNA levels but had no effect on *Sod2* and *Zo1* mRNAs. In addition, none of the treatments had any effect on *Ocln* mRNA levels (Fig. 4A). We also measured the protein levels and found expression patterns consistent with those of the mRNA levels (Fig. 4B and C; Fig. S1D, online only). We then evaluated SOD2 activity in IECs, finding an activity pattern consistent with its mRNA level. This further confirmed that HSCT with increased *Sod2* expression did not affect maternal diabetes–mediated SOD2 suppression in IECs (Fig. 4D). We also measured CLDN1 protein level by immunostaining in IECs and found a pattern consistent with its mRNA level, indicating that STZ‐HSCT/STZ/↑Sod2 treatment reverses, while CTL‐HSCT/CTL/shSod2 treatment mimics, maternal diabetes–mediated CLDN1 suppression in IECs (Fig. 4E and F).

### Transplantation of *Sod2*‐expressing HSCs ameliorates, while *shSod2*‐expressing HSCs mimic, maternal diabetes–mediated oxidative stress in IECs

**Figure 5:**
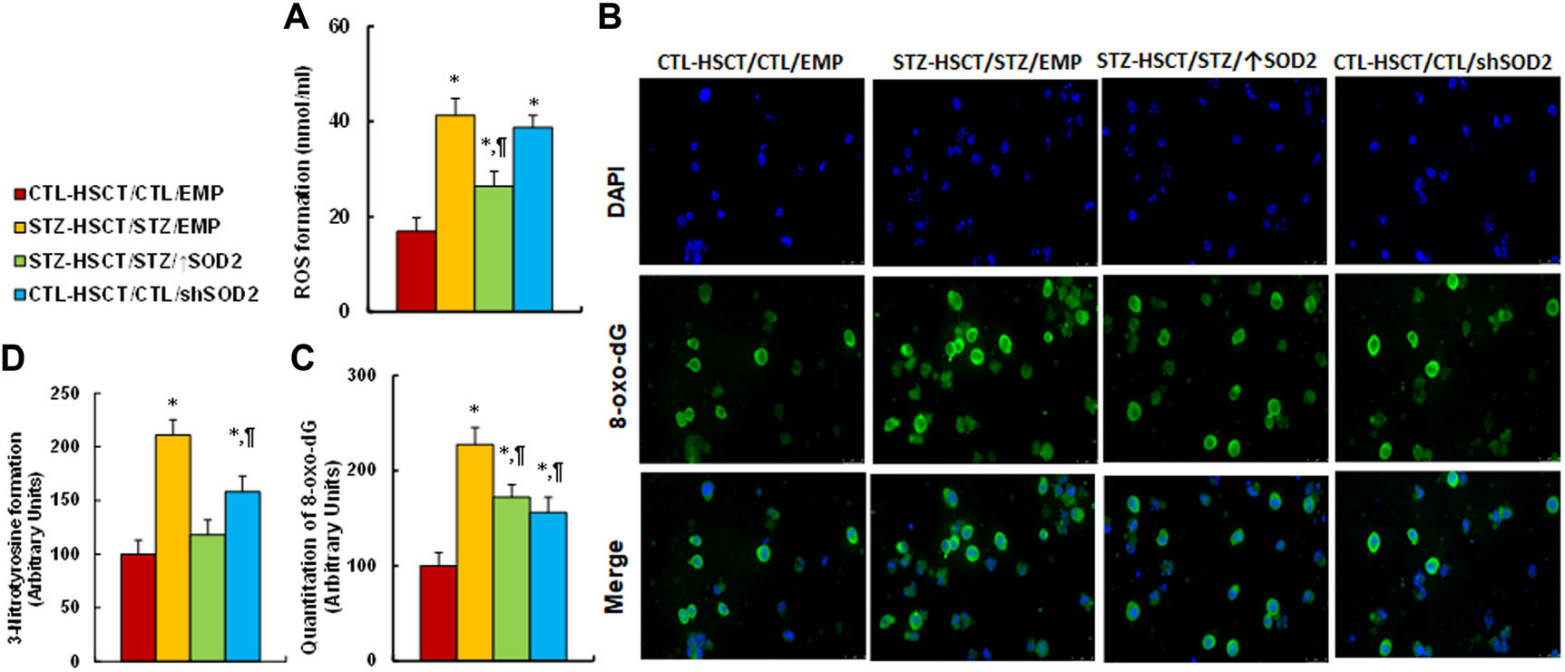
Transplantation of *Sod2*‐expressed HSCs ameliorates, while transplantation of *shSod2*‐expressing HSCs mimics, maternal diabetes–mediated oxidative stress in IECs. Male offspring from either CTL or STZ dams received HSCT with HSCs infected by either *Sod2*‐ or *shSod2*‐expressing lentivirus and then IECs were isolated for biomedical analysis. (A) ROS formation, *n* = 5. (B) 3‐nitrotyrosine formation, *n* = 5. (C) Quantitation of 8‐oxo‐dG formation, *n* = 5. (D) Representative pictures of C for 8‐oxo‐dG staining (green) and DAPI staining for nuclei (blue). ^*^ *P* < 0.05 versus CTL‐HSCT/CTL/EMP group; ^¶^ *P* < 0.05 versus STZ‐HSCT/STZ/EMP group. Data are expressed as mean ± SD.

We then evaluated the potential effect of HSCT and *Sod2* expression on maternal diabetes–mediated oxidative stress in IECs. The results showed that maternal diabetes induction (STZ‐HSCT/STZ/EMP) significantly increased formation of ROS (Fig. 5A), 3‐nitrotyrosine (Fig. 5B), and 8‐oxo‐dG (Fig. 5C and D), compared with the control (CTL‐HSCT/CTL/EMP) group. STZ‐HSCT/STZ/↑Sod2 treatment partly reversed, while CTL‐HSCT/CTL/shSod2 treatment partly mimicked, this effect.

### Transplantation of *Sod2*‐expressing HSCs ameliorates, while *shSod2*‐expressing HSCs mimic, maternal diabetes–mediated inflammation in IECs

Next, we also evaluated the potential effect of HSCT and *Sod2* expression on maternal diabetes–mediated inflammation in IECs. We first determined mRNA levels of the proinflammatory cytokines IL‐1β, IL‐6, MCP1, and IL‐17A. We found that maternal diabetes induction (STZ‐HSCT/STZ/EMP) significantly increased mRNA levels of the cytokines compared with the control (CTL‐HSCT/CTL/EMP) group, and that STZ‐HSCT/STZ/↑Sod2 treatment partly reversed, while CTL‐HSCT/CTL/shSod2 treatment partly mimicked, this effect (Fig. S4A, online only). We also evaluated the secretion of IL‐1β (Fig. S4B, online only), IL‐6 (Fig. S4C, online only), MCP1 (Fig. S4D, online only), and IL‐17A (Fig. S4E, online only) from IECs, and found a pattern consistent with their mRNA levels, indicating that HSCT with increased *Sod2* expression ameliorates maternal diabetes–mediated inflammation in IECs.

### Transplantation of *Sod2*‐expressing HSCs ameliorates, while *shSod2*‐expressing HSCs mimic, maternal diabetes–mediated GI symptoms

**Figure 6:**
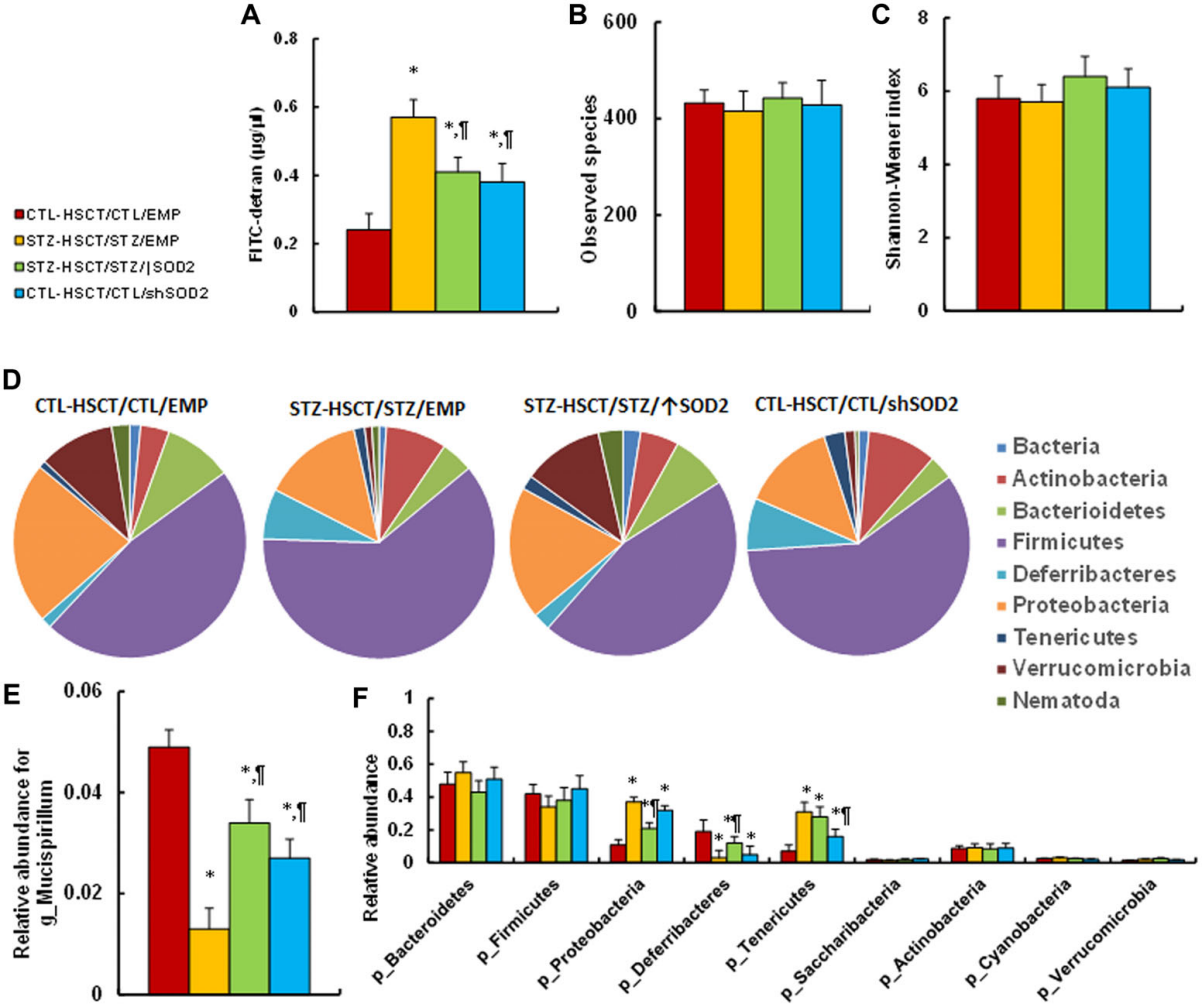
Transplantation of *Sod2*‐expressed HSCs ameliorates, while transplantation of *shSod2*‐expressing HSCs mimics, maternal diabetes–mediated GI symptoms. Male offspring from either CTL or STZ dams received HSCT with HSCs infected by either *Sod2*‐ or *shSod2*‐expressing lentivirus, and then the subsequent recipient offspring were used for analysis of GI symptoms. (A) Intestinal permeability assay by FITC‐dextran, *n* = 5. Gut microbiota analysis, *n* = 9: (B) species richness; (C) species diversity; (D) overview of the identified relative frequencies of different phyla found in treated mice; (E) relative abundance of *Mucispirillumn*; and (F) relative abundance of different bacteria at the phylum level. ^*^ *P* < 0.05 versus CTL‐HSCT/CTL/EMP group; ^¶^ *P* < 0.05 versus STZ‐HSCT/STZ/EMP group. Data are expressed as mean ± SD.

We then evaluated the potential effect of HSCT and *Sod2* expression on maternal diabetes–mediated GI dysfunction. The results showed that maternal diabetes induction (STZ‐HSCT/STZ/EMP) significantly increased intestinal permeability compared with the control (CTL‐HSCT/CTL/EMP) group; STZ‐HSCT/STZ/↑Sod2 treatment partly reversed, while CTL‐HSCT/CTL/shSod2 treatment partly mimicked, this effect (Fig. 6A). We evaluated the gut microbiota using 16S rRNA gene sequencing and found that the various treatments had no effect on microbial species richness (Fig. 6B) and diversity (Fig. 6C). On the other hand, maternal diabetes induction (STZ‐HSCT/STZ/EMP) significantly altered gut microbial composition, compared with the control (CTL‐HSCT/CTL/EMP) group; in particular, the phyla Firmicutes, Proteobacteria, and Verrucomicrobia comprised most of the microbiome in control (CTL‐HSCT/CTL/EMP) mice, while a shift toward Firmicutes occurred in STZ‐HSCT/STZ/EMP diabetic mice, with Verrucomicrobia almost disappearing in the latter. Again, STZ‐HSCT/STZ/↑Sod2 treatment partly reversed, while CTL‐HSCT/CTL/shSod2 treatment partly mimicked, the maternal diabetes–mediated microbiota changes (Fig. 6D). We also determined the relative abundance of different gut organisms and found that maternal diabetes induction (STZ‐HSCT/STZ/EMP) significantly decreased genus *Mucispirillum* (g_Mucispirillum) (Fig. 6E) and increased the phyla Proteobacteria (p_Proteobacteria) and Tenericutes (p_Tenericutes), while it reduced the phylum Deferribacteres (p_Deferribacteres), compared with the control (CTL‐HSCT/CTL/EMP) group (Fig. 6F). Again, STZ‐HSCT/STZ/↑Sod2 treatment partly reversed, while CTL‐HSCT/CTL/shSod2 treatment partly mimicked, this effect. We concluded that HSCT with increased *Sod2* expression ameliorates maternal diabetes–mediated GI dysfunction, including increased intestinal permeability and alteration of gut microbiota.

### Transplantation of *Sod2*‐expressing HSCs ameliorates, while *shSod2*‐expressing HSCs mimic, maternal diabetes–mediated ALB

**Figure 7:**
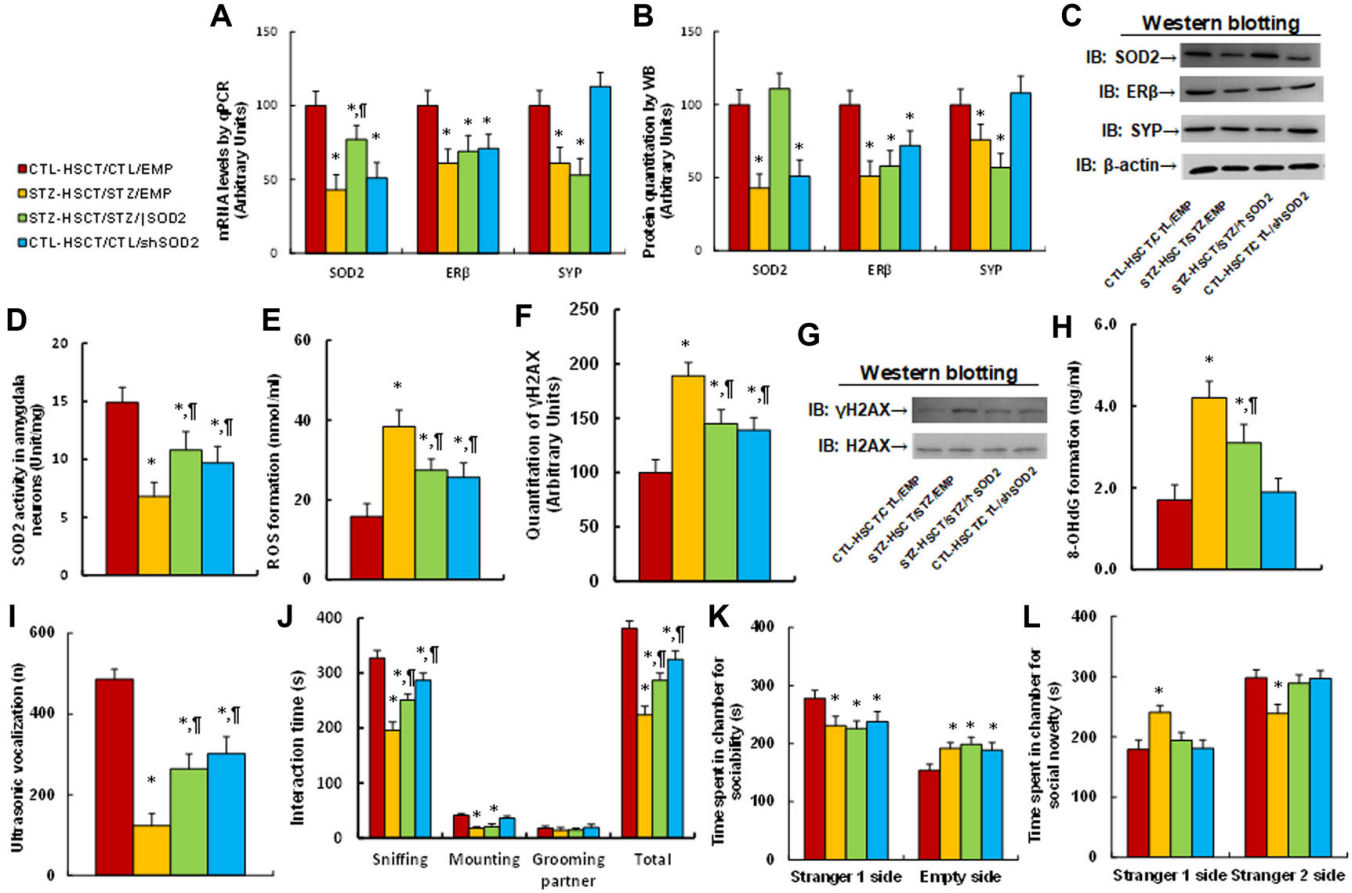
Transplantation of *Sod2*‐expressing HSCs ameliorates, while transplantation of *shSod2*‐expressing HSCs mimics, maternal diabetes–mediated ALB. Male offspring from either CTL or STZ dams received HSCT operation with HSC cells that were infected by either *Sod2*‐ or *shSod2*‐expressing lentivirus, and the subsequent recipient offspring were used for further analysis. The amygdala tissues from recipient offspring were isolated for biomedical analysis: (A) mRNA levels by qPCR, *n* = 4. (B) Quantitation of protein levels, *n* = 5. (C) Representative immunoblots for B. (D) SOD2 activity, *n* = 5. (E) ROS formation, *n* = 5. (F) Quantitation of γH2AX formation, *n* = 5. (G) Representative immunoblots for F. (H) 8‐OHdG formation, *n* = 5. The recipient offspring were used for analysis of ALB: (I) ultrasonic vocalization, *n* = 9. (J) Social interaction (SI) test, with total interaction time and amount of time spent following, mounting, grooming, and sniffing any body parts of the other mouse were calculated, *n* = 9. Three‐chambered social tests, *n* = 9: (K) time spent in chamber for sociability. (L) Time spent in chamber for social novelty. ^*^ *P* < 0.05 versus CTL‐HSCT/CTL/EMP group; ^¶^ *P* < 0.05 versus STZ‐HSCT/STZ/EMP group. Data are expressed as mean ± SD.

We evaluated the potential effect of HSCT with increased *Sod2* on maternal diabetes–mediated ALB. We first measured gene expression in amygdala tissues and found that maternal diabetes induction (STZ‐HSCT/STZ/EMP) significantly decreased the mRNA levels of *Sod2*, *Esr2* (ERβ), and *Syp* compared with the control (CTL‐HSCT/CTL/EMP) group. STZ‐HSCT/STZ/↑Sod2 treatment partly reversed, while CTL‐HSCT/CTL/shSod2 treatment mimicked, the effect on mRNAs of *Sod2* and *Esr2*, while it had no effect on *Syp* mRNA (Fig. 7A). We also evaluated protein levels and found a pattern consistent with the mRNA levels (Fig. 7B and C; Fig. S1E, online only). Furthermore, we evaluated SOD2 activity; again, STZ‐HSCT/STZ/↑Sod2 treatment partly reversed, while CTL‐HSCT/CTL/shSod2 treatment mimicked, maternal diabetes–mediated *Sod2* suppression (Fig. 7D). In addition, we measured gene expression in other brain tissues. We found that maternal diabetes induction (STZ‐HSCT/STZ/EMP) significantly decreased SYP levels in the hypothalamus but had no effect on SOD2 and ERβ, compared with the control (CTL‐HSCT/CTL/EMP) group; the STZ‐HSCT/STZ/↑Sod2 and CTL‐HSCT/CTL/shSod2 groups showed no effect (Fig. S5A, online only). Additionally, mRNA levels in the hippocampus showed no difference among the treatments (Fig. S5B, online only).

We then evaluated the effect on oxidative stress and found that maternal diabetes induction (STZ‐HSCT/STZ/EMP) significantly increased the formation of ROS (Fig. 7E), γH2AX (Fig. 7F and G; Fig. S1F, online only), and 8‐OHdG (Fig. 7H), compared with the control (CTL‐HSCT/CTL/EMP) group. STZ‐HSCT/STZ/↑Sod2 treatment partly reversed, while CTL‐HSCT/CTL/shSod2 treatment partly mimicked, this effect.

Finally, we determined ALB in the offspring mice, and the results showed that maternal diabetes (STZ‐HSCT/STZ/EMP) treatment significantly decreased USVs (Fig. 7I). Additionally, offspring from the STZ‐HSCT/STZ/EMP group spent significantly less time sniffing, mounting, and interacting in total with stranger mice during the SI tests (Fig. 7J) compared with the CTL‐HSCT/CTL/EMP group. STZ‐HSCT/STZ/↑Sod2 treatment partly reversed, while CTL‐HSCT/CTL/shSod2 treatment partly mimicked, this effect, but neither treatment influenced mounting interaction time. Furthermore, offspring from the maternal diabetes–induced (STZ‐HSCT/STZ/EMP) group showed significantly less interest in sociability (Fig. 7K) and social novelty (Fig. 7I) compared with the control (CTL‐HSCT/CTL/EMP) group; STZ‐HSCT/STZ/↑Sod2 treatment showed no effect, while CTL‐HSCT/CTL/shSod2 treatment mimicked this effect. We conclude that HSCT with increased *Sod2* expression ameliorates maternal diabetes–mediated ALB.

### Schematic model for the potential effect of HSCT with increased *Sod2* expression on maternal diabetes–mediated GI symptoms and ALB

**Figure 8:**
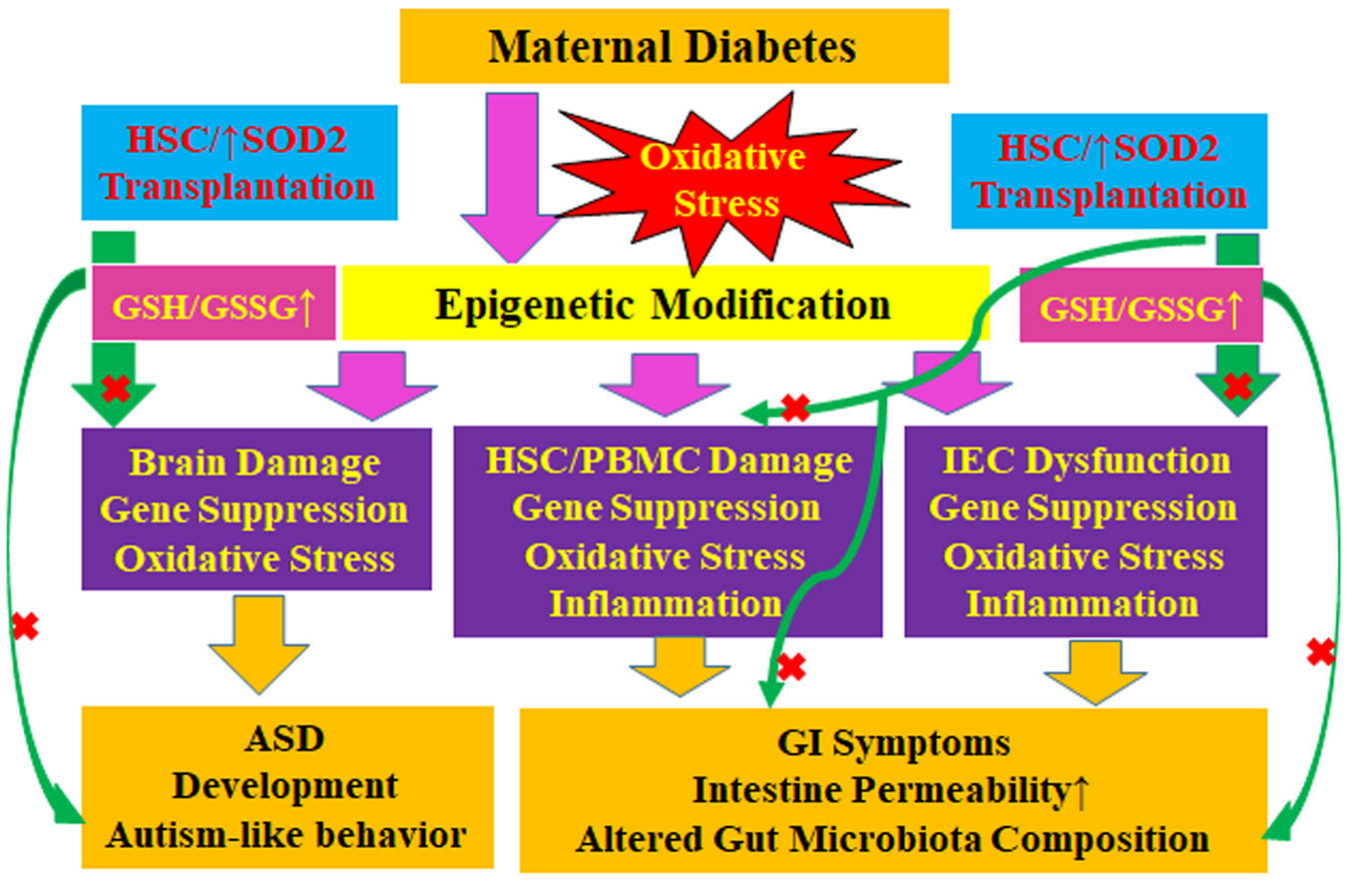
Schematic model for the potential effect of HSCT on maternal diabetes–mediated GI symptoms and ALB. ASD, autism spectrum disorder; GI, gastrointestinal; GSH, reduced glutathione; GSSG, oxidized glutathione; HSCs, hematopoietic stem cells; IEC, intestine epithelial cell; PBMCs, peripheral blood mononuclear cells; SOD2, superoxide dismutase 2.

Maternal diabetes–mediated oxidative stress triggers epigenetic modifications in subsequent offspring tissues. Epigenetic modification in IECs and HSCs/PBMCs results in gene suppression, oxidative stress, and inflammation, subsequently contributing to GI symptoms, which include increased intestinal permeability and altered gut microbiota compositions. Furthermore, epigenetic modification in the brain results in gene suppression and oxidative stress, triggering ASD development and ALB. On the other hand, HSCT operation and the manipulation of *Sod2* expression diminish maternal diabetes–mediated systematic oxidative stress (e.g., increased GSH/GSSG ratio), subsequently reversing maternal diabetes–mediated GI symptoms and ASD development (Fig. 8).

## Discussion

In this study, we found that transient hyperglycemia induces persistent suppression of tight junction proteins by epigenetic modifications. Maternal diabetes induces gene suppression, oxidative stress, and inflammation in PBMCs and IECs, subsequently contributing to GI dysfunction in autism‐like animal models, while HSCT with increased *Sod2* expression partly reverses this effect and subsequently ameliorates maternal diabetes–mediated ALB.

### Potential effect of HSCT on maternal diabetes–mediated IECs and GI dysfunction

It has been reported that hyperglycemia‐mediated ROS overgeneration is the potential driving force for diabetic complications,^43^ and transient hyperglycemia triggers persistent epigenetic changes by ROS generation.^30^ We have recently reported that maternal diabetes induces ALB through hyperglycemia‐mediated persistent oxidative stress and *Sod2* mRNA suppression, indicating that SOD2 may play an important role in maternal diabetes–mediated ALB and GI dysfunction.^5^ In our study here, we found that transient hyperglycemia induced persistent suppression of tight junction proteins, including ZO1 and CLDN1, in human epithelial cells due to epigenetic changes. Furthermore, maternal diabetes induced suppression of tight junction proteins in autism‐like offspring in IECs, as well as GI dysfunction with increased intestinal permeability and altered microbiota compositions. On the other hand, transplantation of *Sod2*‐expressing HSCs partly reversed, while transplantation of *shSod2*‐expressing HSCs partly mimicked this effect, indicating that maternal diabetes–mediated oxidative stress and subsequent epigenetic changes and gene suppression could be the potential driving force for GI dysfunction in autism‐like offspring of female mice with maternal diabetes. Also, interestingly, we found that transplantation of *Sod2*‐expressing HSCs could partly reverse maternal diabetes–mediated *Cldn1* suppression in IECs in offspring, which can be explained by *Sod2*‐expressing HSCs reducing maternal diabetes–mediated ROS generation, subsequently reversing oxidative stress–mediated epigenetic changes on the *Cldn1* promoter.^4^, ^6^ In addition, the antioxidant and SOD mimetic MnTBAP^5^ was also used for the treatment and produced a similar effect as SOD2 on IECs, PBMCs, and GI dysfunction, but had little effect on maternal diabetes–mediated ALB; one explanation for this is that antioxidants may be effective transiently, while *Sod2* expression is not.^43^

### HSCs can differentiate into PBMCs after HSCT

We have previously found that maternal diabetes–mediated oxidative stress triggers epigenetic changes and gene suppression in both HSCs and PBMCs. PBMCs can inherit the similar properties from HSCs during differentiation after HSCT operation, and these properties may include epigenetic changes and subsequent gene suppression.^25^ In this study, we showed that GFP lentivirus–infected HSCs from donor mice were found in PBMCs from recipient mice with ∼16.3% GFP^+^ after HSCT; this further shows that HSCs can differentiate into PBMCs after HSCT, even though the number of transduced cells is relatively low. This can be explained by the following: (1) GFP lentivirus is lost during HSC differentiation in the absence of selective antibiotics; in fact, most of PBMCs differentiate after HSCT and are not labeled by GFP; (2) GFP^+^ HSCs are partly removed by the recipient immune system after HSCT; and/or (3) GFP^+^ HSCs partly lost their function owing to the presence of GFP protein interference. We assume that HSCs used for HSCT in autism‐like offspring may have a higher chance of survival and function because of the absence of GFP interference.^34^

### Potential effect of HSCT on maternal diabetes–mediated ALB

We have previously reported that amygdala neurons play a dominant role in maternal diabetes–mediated ALB through oxidative stress–mediated epigenetic changes and subsequent gene suppression in autism‐like offspring.^4^, ^5^, ^6^ In our study here, we found that transplantation of *Sod2*‐expressing HSCs partly reversed both maternal diabetes–mediated *Sod2* suppression in amygdala tissues as well as subsequent oxidative stress and ALB in offspring. A remaining question is how HSCT affects maternal diabetes–mediated ALB in the presence of the blood brain barrier.^18^ It has been reported that increased oxidative stress in the plasma, especially increased GSH/GSSG ratio, can be the potential biomarker for ASD subjects;^44^, ^45^ our results above have shown that transplantation of *Sod2*‐expressing HSCs partly reversed the maternal diabetes–mediated GSH/GSSG ratio increase in serum. We hypothesize that HSCT with increased *Sod2* expression diminishes maternal diabetes–mediated systematic oxidative stress and epigenetic changes in neurons, subsequently ameliorating ALB in autism‐like offspring.

## Conclusions

Maternal diabetes induces persistent epigenetic changes and gene suppression in PBMCs, IECs, and neurons, while transplantation of HSCs ameliorates maternal diabetes–mediated GI dysfunction and ALB by systematically reversing maternal diabetes–mediated oxidative stress. HSCs may play a potential therapeutic role for clinical treatment of Individuals with ASD.

## Competing interests

The authors declare no competing interests.

## Ethical approval and consent to participate

The animal protocol conformed to U.S. NIH guidelines (Guide for the Care and Use of Laboratory Animals, No. 85‐23, revised 1996), and was reviewed and approved by the Institutional Animal Care and Use Committee from Foshan Maternity and Child Healthcare Hospital.

## Author contributions

P.Y. wrote the paper. P.Y., H.Y., and J.L. designed, analyzed the data, and interpreted the experiments. R.S. and S.H. performed part of the mouse experiments. Z.W. and L.X. performed part of the gene analysis. J.Z. and Y.L. performed the remaining experiments. All authors read and approved the final manuscript.

### Peer review

The peer review history for this article is available at https://publons.com/publon/10.1111/nyas.14766.

## Supporting information


**Figure S1**. Representative pictures of full immunoblots.

**Figure S2**. Potential effect of transient hyperglycemia on epigenetic modifications on the *Cldn1* promoter.

**Figure S3**. Potential effect of transient hyperglycemia on epigenetic modifications on the *Zo1* promoter.

**Figure S4**. Transplantation of *Sod2*‐expressing HSCs ameliorates, while transplantation of *shSOD2*‐expressing HSCs mimics, maternal diabetes‐mediated inflammation in IEC.

**Figure S5**. Potential effect of HSCT on maternal diabetes–mediated gene expression in the brain.

**Table S1**. Sequences of primers for real‐time quantitative PCR (qPCR)

**Supplementary File S1**. Materials and methods
