# Supplementary material for: Hematopoietic stem cell transplantation ameliorates maternal diabetes–mediated gastrointestinal symptoms and autism‐like behavior in mouse offspring
Source: Ann N Y Acad Sci. 2022 Feb 27;1512(1):98–113. doi: 10.1111/nyas.14766 (PMC9307016; doi:10.1111/nyas.14766)
Supplement: Supplementary file 1 — Figure S1. Representative pictures of full immunoblots. [file NYAS-1512-98-s002.docx]

FIGURE S1

**Figure S1. Representative pictures of full blots for Western Blotting.** (a) Full blots for Figure 1c. (b) Full blots for Figure 2e. (c). Full blots for Figure 2j. (d) Full blots for Figure 4c. (e) Full blots for Figure 7c. (f). Full blots for Figure 7g.
