# Supplementary material for: Hematopoietic stem cell transplantation ameliorates maternal diabetes–mediated gastrointestinal symptoms and autism‐like behavior in mouse offspring
Source: Ann N Y Acad Sci. 2022 Feb 27;1512(1):98–113. doi: 10.1111/nyas.14766 (PMC9307016; doi:10.1111/nyas.14766)
Supplement: Supplementary file 2 — Figure S2. Potential effect of transient hyperglycemia on epigenetic modifications on the Cldn1 promoter. [file NYAS-1512-98-s006.docx]

FIGURE S2

**Figure S2. Potential effect of transient hyperglycemia on epigenetic modifications on the CLDN1 promoter.** Human Colon Stem Cells were treated with either 5mM low glucose (LG) or 25mM high glucose (HG) for 4 days. The cells were then infected by control (CTL), SOD2 overexpression (↑SOD2), or SOD2 knockdown (shSOD2) lentivirus for one day before they were then treated by LG for another 4 days in the presence of 1% serum; the cells were then harvested for ChIP analysis. (a) Histone H4 methylation on the CLDN1 promoter, n=4. (b) Histone acetylation on the CLDN1 promoter using H3K9,14,18,23,27ac and H4K5,8,12,16ac antibodies, n=4. Data were expressed as mean ± SEM.
