# Supplementary material for: Hematopoietic stem cell transplantation ameliorates maternal diabetes–mediated gastrointestinal symptoms and autism‐like behavior in mouse offspring
Source: Ann N Y Acad Sci. 2022 Feb 27;1512(1):98–113. doi: 10.1111/nyas.14766 (PMC9307016; doi:10.1111/nyas.14766)
Supplement: Supplementary file 4 — Figure S4. Transplantation of Sod2‐expressing HSCs ameliorates, while transplantation of shSOD2‐expressing HSCs mimics, maternal diabetes‐mediated inflammation in IEC. [file NYAS-1512-98-s004.docx]

FIGURE S4

**Figure S4. Transplantation of SOD2 expressed HSC ameliorates, while transplantation of SOD2 knockdown HSC mimics, maternal diabetes-mediated inflammation in IEC**. Male offspring from either CTL or STZ dams received HSCT operation with HSC cells that infected by either SOD2 or shSOD2 lentivirus, and the IEC were isolated for analysis of proinflammatory cytokine release. (a) mRNA levels of pre-inflammatory cytokines, n=4. (b) IL-1β secretion, n=9. (c) IL-6 secretion, n=9. (d) MCP1 secretion, n=9. (e) IL17A secretion, n=9. *, *P*<0.05, vs. CTL-HSCT/CTL/EMP group; ¶, *P*<0.05, vs. STZ-HSCT/STZ/EMP group. Data were expressed as mean ± SEM.
