# Supplementary material for: Hematopoietic stem cell transplantation ameliorates maternal diabetes–mediated gastrointestinal symptoms and autism‐like behavior in mouse offspring
Source: Ann N Y Acad Sci. 2022 Feb 27;1512(1):98–113. doi: 10.1111/nyas.14766 (PMC9307016; doi:10.1111/nyas.14766)
Supplement: Supplementary file 5 — Figure S5. Potential effect of HSCT on maternal diabetes–mediated gene expression in the brain. [file NYAS-1512-98-s003.docx]

FIGURE S5

**Figure S5.** **Potential effect of** **HSCT on maternal diabetes-mediated gene expression in the brain**. Male offspring from either CTL or STZ dams received HSCT operation with HSC cells that infected by either SOD2 or shSOD2 lentivirus, and the tissues for hypothalamus and hippocampus were isolated from subsequent recipient offspring for mRNA analysis. (a) mRNA levels in hypothalamus, n=4. (b) mRNA levels in hippocampus, n=4. *, *P*<0.05, vs CTL/VEH group. Data were expressed as mean ± SEM.
