# Supplementary material for: Hematopoietic stem cell transplantation ameliorates maternal diabetes–mediated gastrointestinal symptoms and autism‐like behavior in mouse offspring
Source: Ann N Y Acad Sci. 2022 Feb 27;1512(1):98–113. doi: 10.1111/nyas.14766 (PMC9307016; doi:10.1111/nyas.14766)
Supplement: Supplementary file 6 — Table S1. Sequences of primers for real‐time quantitative PCR (qPCR) [file NYAS-1512-98-s005.docx]

**Table S1. Sequences of primers for the real time quantitative PCR (qPCR)**

| Gene | Species | Analysis | Forward primer (5'→3') | Reverse primer (5'→3') |
| --- | --- | --- | --- | --- |
| β-actin | Human | mRNA | gatgcagaaggagatcactgc | atactcctgcttgctgatcca |
| CLDN1 | Human | mRNA | agatgaggatggctgtcattg | agcctgaccaaattcgtacct |
| OCLN | Human | mRNA | tgattcggatcctgtctatgc | agctaccaaagccacttcctc |
| RORA | Human | mRNA | ggagaagtcagcaaagcaatg | gacattcggccaaattttaca |
| SOD2 | Human | mRNA | gcctacgtgaacaacctgaac | tgaggtttgtccagaaaatgc |
| ZO1 | Human | mRNA | cgtgctgacttctggagattc | ggttacaggcctcagaaatcc |
| CLDN1 | Human | ChIP | aaaactgcagctcttgaagga | tcgctttctctcgtggatct |
| ZO1 | Human | ChIP | gaggagcaagtggacaagtctc | agccgggtaacccaagtaac |
| β-actin | Mouse | mRNA | tcttgggtatggaatcctgtg | atctccttctgcatcctgtca |
| CLDN1 | Mouse | mRNA | caatgccaggtatgaatttgg | cactagaaggtgttggcttgg |
| ERβ | Mouse | mRNA | atgtgctatggccaacttctg | caagcttcctcttcagggtct |
| OCLN | Mouse | mRNA | ccatctgactatgcggaaaga | ccacactcaaggtcagaggaa |
| RORA | Mouse | mRNA | attggacatcaatgggatcaa | tttggatatgttctgggcaag |
| SYP | Mouse | mRNA | ttcgctttcatgtggctagtt | aagtcacagggtccctcagtt |
| SOD2 | Mouse | mRNA | ggcctacgtgaacaatctcaa | tcaggtttgtccagaaaatgg |
| ZO1 | Mouse | mRNA | tagacgatcatccacccaaag | attaggcagagcaccatcaga |
