# Supplementary material for: Hematopoietic stem cell transplantation ameliorates maternal diabetes–mediated gastrointestinal symptoms and autism‐like behavior in mouse offspring
Source: Ann N Y Acad Sci. 2022 Feb 27;1512(1):98–113. doi: 10.1111/nyas.14766 (PMC9307016; doi:10.1111/nyas.14766)
Supplement: Supplementary file 7 — Supplementary File S1. Materials and methods [file NYAS-1512-98-s007.docx]

**Supplementary File S1. Materials and methods**

**Reagents and materials**. The QualiCell® Human Colon Stem Cells -XLC401 (#CSC-C00314) and related maintenance media (#CM-1339Z) were obtained from Creative Bioarray (Beijing, China). All cells were maintained in a humidified incubator with 5% CO_2_ at 37°C.

The antibodies for β-actin (sc-47778), CLDN1 (sc-166338), OCLN (sc-133256), ERβ (sc-390243), SOD2 (sc-30080) and ZO1 (sc-33725) were obtained from Santa Cruz Biotechnology. The antibody for 8-oxo-dG (4354-MC-050) was purchased from Novus Biologicals; Antibodies for acetyl-histone H4 K5, K8, K12, and K16 (H4K5,8,12,16ac, #PA5-40084) were obtained from Invitrogen. Antibodies for histone H3 acetyl K9, K14, K18, K23, K27(H3K9,14,18,23,27ac, ab47915), H4K20me1 (ab9051), H4K20me3 (ab9053), H4R3me1 (ab17339), H3K9me2 (ab1220), H3K9me3 (ab8898), H3K27me2 (ab24684), H3K27me3 (ab6002), H2AX (ab20669) and γH2AX (ab2893) were obtained from Abcam, and 3-nitrotyrosine (3-NT) was measured using the 3-Nitrotyrosine ELISA Kit (ab116691 from Abcam) per manufacturers’ instructions. The mitochondrial fraction was isolated using a Pierce Mitochondria Isolation Kit (Pierce Biotechnology) per manufacturers’ instructions. Nuclear extracts were prepared using the NE-PER Nuclear and Cytoplasmic Extraction Reagents Kit (Pierce Biotechnology). Protein concentration was measured using the Coomassie Protein Assay Kit (Pierce Biotechnology). Fluorescein isothiocyanate-labeled dextran (FITC-dextran, #46944) and streptozocin (STZ, #18883-66-4) were obtained from Sigma.

**Preparation of SOD2 expression lentivirus***.* The human SOD2 expression lentivirus was prepared previously in our lab (1). The cDNA for mouse SOD2 (obtained from Open Biosystems) was subcloned into the pLVX-Puro vector (from Clontech) with the underlined restriction sites using the following primers: SOD2 forward primer: 5’- gtac- ctcgag- atg ttg tgt cgg gcg gcg tgc-3’ (Xho1) and SOD2 reverse primer: 5’- gtac- tctaga- tca ctt ctt gca agc tgt gta -3’ (Xba1). The lentivirus for SOD2 or empty control (CTL) was expressed through Lenti-X™ Lentiviral Expression Systems (from Clontech) per manufacturers’ instructions, and the prepared lentivirus was used for infection of cells for the expression of either empty (EMP) or SOD2 (↑SOD2).

**Preparation of shSOD2 knockdown lentivirus**. The human shSOD2 lentivirus was prepared previously in our lab (1). The shRNA lentivirus plasmid for mouse SOD2 (sc-41656-SH) or non-target control (sc-108060) were purchased from Santa Cruz Biotechnology. The related lentivirus for shSOD2 or empty control (CTL) were expressed through Lenti-X™ Lentiviral Expression Systems (from Clontech) per manufacturers’ instructions. The purified and condensed lentivirus was used for *in vitro* gene knockdown, and knockdown efficiency was confirmed by mRNA reduction of more than 65% compared to the control group using real time PCR (see Table S1).

**Preparation of GFP lentivirus particles for HSC infection.** The pLenti-GFP Lentiviral Control Vector (#LTV-400) and related products were obtained from Cell Biolabs Inc. The lentiviral supernatant was produced by cotransfecting 293T cells (#LTV-100) with pLenti-GFP and the ViraSafe™ Lentiviral Packaging System (#VPK-206). The lentivirus was concentrated and purified using the ViraBind™ Lentivirus Concentration and Purification Kit (#VPK-090), and the virus was used to infect isolated HSC cells using the ViraDuctin™ Lentivirus Transduction Kit (#LTV-200) (2).

**RT reaction and real-time quantitative PCR**. Total RNA from treated cells was extracted using the RNeasy Micro Kit (Qiagen), and the RNA was reverse transcribed using an Omniscript RT kit (Qiagen). All the primers were designed using Primer 3 Plus software with the Tm at 60°C, primer size of 21 bp, and product length in the range of 140-160 bp (see Table S1). The primers were validated with an amplification efficiency in the range of 1.9-2.1 and the amplified products were confirmed with agarose gel. Real-time quantitative PCR was run on iCycler iQ (Bio-Rad) with the Quantitect SYBR green PCR kit (Qiagen). PCR was performed by denaturing at 95°C for 8 min followed by 45 cycles of denaturation at 95°C, annealing at 60°C, and extension at 72°C for 10 s, respectively. 1 µl of each cDNA was used to measure target genes. β-actin was used as the housekeeping gene for transcript normalization, and the mean values were used to calculate relative transcript levels with the ^ΔΔ^CT method per instructions from Qiagen. In brief, the amplified transcripts were quantified by the comparative threshold cycle method using β-actin as a normalizer. Fold changes in gene mRNA expression were calculated as 2^−ΔΔCT^ with CT = threshold cycle, ΔCT=CT (target gene)-CT(β-actin), and the ΔΔCT=ΔCT (experimental)-ΔCT (reference).

**Western blotting.** Cells were lysed in an ice-cold lysis buffer (0.137M NaCl, 2mM EDTA, 10% glycerol, 1% NP-40, 20mM Tris base, pH 8.0) with protease inhibitor cocktail (Sigma). The proteins were separated in 10% SDS-PAGE and transferred to the PVDF membrane, which was then blotted using primary antibodies (1:1000) and then simultaneously incubated with the differentially labeled species-specific secondary antibodies, anti-RABBIT IRDye™ 800CW (green) and anti-MOUSE (or goat) ALEXA680 (red). Membranes were scanned and quantitated using the ODYSSEY Infrared Imaging System (LI-COR, NE) (3).

**Chromatin immunoprecipitation (ChIP).** Cells were washed and crosslinked using 1% formaldehyde for 20 min and terminated by 0.1 M glycine. Cell lysates were sonicated and centrifuged, and 500 µg of protein were pre-cleared by BSA/salmon sperm DNA with preimmune IgG and a slurry of Protein A Agarose beads. Immunoprecipitations were performed with the indicated antibodies, BSA/salmon sperm DNA and a 50% slurry of Protein A agarose beads. Input and immunoprecipitates were washed, eluted, and then incubated with 0.2mg/ml Proteinase K for 2 h at 42˚C, followed by 6 h at 65°C to reverse the formaldehyde crosslinking. DNA fragments were recovered through phenol/chloroform extraction and ethanol precipitation. A ~150 bp fragment on the related promoters was amplified by real-time PCR (qPCR) using the primers provided in Table S1.

**Measurement of ROS generation.** Treated cells were seeded in a 24-well plate and incubated with 10μM CM-H2DCFDA (Invitrogen) for 45 min at 37°C, and then the intracellular formation of reactive oxygen species (ROS) was measured at excitation/emission wavelengths of 485/530nm using a FLx800 microplate fluorescence reader (Bio-Tek). The data was normalized as arbitrary units (4, 5).

**Measurement of DNA breaks.** The 8-hydroxy-2'-deoxyguanosine **(**8-OHdG**)** formation was measured using an OxiSelect™ Oxidative DNA Damage ELISA Kit (Cat No. STA320, from Cell Biolabs Inc.) according to manufacturers’ instructions. The formation of γH2AX was measured from nuclear extracts by western blotting using H2AX as the input control.

**DNA methylation analysis.** We developed a real-time PCR-based method for methylation-specific PCR (MSP) analysis on the human CLDN1/ZO1 promoter according to the previously described method with some modifications (6, 7). Genomic DNA from human cells was extracted and purified before then being treated by bisulfite modification using the EpiJET Bisulfite Conversion Kit (#K1461, Fisher). The modified DNA was then amplified using methylated and unmethylated primers for MSP that were designed using Methprimer software (<http://www.urogene.org/cgi-bin/methprimer/methprimer.cgi>) with the following details:

CLDN1 Methylated primer: forward 5’- ttt ata gga gcg aga aga ttt acg a -3’, reverse 5’- ccc taa cga ttt caa aac gac -3’; CLDN1 unmethylated primer: forward 5’- ttt ata gga gtg aga aga ttt atg a -3’; reverse 5’- ccc cta aca att tca aaa caa c -3’. product size: 152bp (methylated) & 153bp (unmethylated); CpG island size: 172bp; Tm: 66.0°C. The final methylation readout was normalized by unmethylated input PCR.

ZO1 Methylated primer: forward 5’- gtt ttt cgg aga tga aag tta tga c -3’, reverse 5’- taa aaa aac cga caa aac cga t -3’; ZO1 unmethylated primer: forward 5’- agt ttt ttg gag atg aaa gtt atg at -3’; reverse 5’- taa aaa aac caa caa aac caa t -3’. product size: 152bp (methylated) & 153bp (unmethylated); CpG island size: 162bp; Tm: 69.0°C. The final methylation readout was normalized by unmethylated input PCR.

**In vivo mouse experiments.** The animal protocol conformed to US NIH guidelines (Guide for the Care and Use of Laboratory Animals, No. 85-23, revised 1996) and was reviewed and approved by the Institutional Animal Care and Use Committee from Foshan Maternity and Child Health Care Hospital and Kangning Hospital of Shenzhen. In this study, 10 dams were assigned for each exposure, and one representative offspring was picked up randomly from each dam for experiments and analysis, and 9 representative offspring were usually picked up from 10 in total in case animal may die by accident during experimental process. Each animal was firstly used for behavior analysis, then was sacrificed for tissue collection and subsequent biological analysis. Each experimental offspring was in separate cage since they came from different dams.

Protocol 1. Generation of diabetic mice. All the experimental mice with a C57BL/6J mixed genetic background were monitored for estrous cycles with daily vaginal smears. Only mice with at least two regular 4 to 5-day estrous cycles were included in the studies. Chronic diabetic female mice were induced by injection of 35 mg/kg streptozocin (STZ, 0.05 M sodium citrate, pH 5.5) after an 8-hour fasting period. One week after injection, blood glucose was monitored continuously for 3 days and animals with blood glucose >250mg/dl were considered positive. Control (CTL) mice received only vehicle injection. Females from the CTL and STZ groups were caged with males for pregnancy to generate subsequent offspring, and one of offspring from each verified pregnant dams (n=9 for each group) was used for the subsequent treatment in Protocol 3.

Protocol 2. Preparation of hematopoietic stem cells with the manipulation of SOD2 expression. Mouse HSC were isolated from 4-6 week-old male offspring donor mice from either the CTL or STZ group as described below. The cells were then purified by density centrifugation using Histopaque 1083^®^ (#-1083-1, Sigma) and resuspended in 10 ml of RPMI 1640 supplemented with 10% FBS and 2 mM EDTA. The isolated HSCs were infected by lentivirus for the expression of empty (EMP), SOD2 expression (↑SOD2) or knockout (shSOD2) in order to develop HSC/EMP, HSC/↑SOD2 or HSC/shSOD2 cells.

Protocol 3. Postnatal treatment by hematopoietic stem cell transplantation (HSCT). Male offspring (6 weeks old) from either the CTL or STZ group were used as recipients for HSCT. The recipient male mice were lethally irradiated with 2 doses of 6 Gy 3 hours apart (8), and after 4 hours of irradiation, 2×10^6^ of HSC cells prepared from Protocol 2 were systemically transplanted by tail vein injection. All transplant-recipient mice were set aside for a minimum of 12 weeks to allow for complete reconstitution of the bone marrow before they were then used for analysis. The experimental mice were randomly separated into the following 4 groups: Group 1: Offspring from CTL group receiving HSCT with HSC cells from the CTL group that were infected by EMP lentivirus (CTL-HSCT/CTL/EMP); Group 2: Offspring from STZ group receiving HSCT with HSC cells from the STZ group that were infected by EMP lentivirus (STZ-HSCT/STZ/EMP); Group 3: Offspring from STZ group receiving HSCT with HSC cells from the STZ group that were infected by ↑SOD2 lentivirus (STZ-HSCT/STZ/↑SOD2); Group 4: Offspring from CTL group receiving HSCT with HSC cells from the CTL group that were infected by shSOD2 lentivirus (CTL-HSCT/CTL/shSOD2). All transplant-recipient mice were set aside for a minimum of 12 weeks to allow for complete reconstitution of the bone marrow before they were used for evaluation of ALB and GI symptoms, and the brain tissues, including amygdala, hypothalamus and hippocampus, were collected. The whole blood was collected by heart puncture and the serum and peripheral blood mononuclear cells (PBMC) were isolated. The intestine epithelial cells (IEC) were isolated as described below for further biomedical analysis.

**Animal behavior test.** The autism-like behavior test from mouse offspring was evaluated using ultrasonic vocalization (USV), social interaction (SI) tests and a three-chambered social test as described below (9-11).

Ultrasonic vocalizations (USV). The USV of neonates were examined during a 5-min maternal separation paradigm on postnatal day 7. USV from individually-isolated pups were recorded using an externally polarized condenser microphone with a frequency range of 30-300 kHz that was attached 15-20 cm above the floor of an isolation chamber. The microphone was connected to the Avisoft-UltrasoundGate recording software (Avisoft Bioacoustics, Germany) and the pup-emitted calls were recorded to WAV sound files using parameters optimized for mice. Pups were individually placed in the sound-proof chambers and calls were recorded for 300s. Data transformation on the number of USV were analyzed using a generalized linear model with a negative binomial distribution and a log-link function (9, 10).

Social interaction (SI) test. The subjects (Test and Stranger) were separately habituated to the arena for 5 min before the test. The animal used as the “Stranger” was used only once and was a mouse of the same gender, weight, and age that had no previous contact with the test mice (12). During each test, the mice were placed into the apparatus over a period of 20 min and the amount of time spent following, mounting, grooming, and sniffing any body parts of the other mouse was taken as an indicator of social engagement. Social interaction time was calculated and analyzed using EthoVision XT animal tracking software (Noldus, USA).

Three-chambered social test. 7-8 week-old mice were used to assess sociability and preference for social novelty. Target subjects (Stranger 1 and Stranger 2) were 7-8 week-old mice habituated to being placed inside wire cages for 3 days prior to the beginning of testing. Test mice were habituated to the testing room for at least 45 min prior to the start of behavioral tasks. For the sociability test, the test animal was introduced to the middle chamber and left to habituate for 5 min, after which an unfamiliar mouse (Stranger 1) was introduced into a wire cage in one of the side-chambers and an empty wire cage on the other side-chamber. The test animal was allowed to freely explore all 3 chambers over a 10 min session. Following this, a novel stranger mouse (Stranger 2) was introduced into the previously empty wire cage and the test animal was again left to explore for a 10 min session. Parameters scored include time spent in each chamber and number of entries into the chambers. Time spent in each chamber and track maps were calculated using automated SMART software (1, 11).

**SOD2 activity assay.** SOD2 was obtained from the mitochondrial fraction that was isolated using a Pierce Mitochondria Isolation Kit (Pierce) according to manufacturers’ instructions. SOD activity was measured as described previously (13). In brief, a stable O2.- source was generated through the conversion action of XOD (xanthine oxidase) from xanthine and was mixed with chemiluminescent (CL) reagents to achieve a stable light emission. The SOD2 sample injection can scavenge O2.- and the subsequent decrease of chemiluminescent response is proportional to SOD2 activity. This system can have a detection limit of 0.001U/ml with the linear range of 0.03~2.00 U/ml. The results were normalized by protein concentration and expressed as Units/mg proteins (U/mg) (14).

**Isolation and characterization of hematopoietic stem cells (HSC)**. The HSC preparation procedure is a minor modification from previously described method (15). In brief, the whole bone marrow cells were collected from tibias in treated mice. Bone marrow cells were stained with antibodies for the identification of HSC (c-Kit^+^/Sca1^+^/Lineage^-^), and the following antibodies were used: c-Kit-PE (#12-1171-82), Sca-1-FITC (#11-5981-82), and anti-Lineage Antibody Cocktail comprises a mixture of PE-Cy5-conjugated antibodies, including anti-B220, anti-CD4, anti-CD8, anti-Gr-1, anti-Mac-1, and anti-TER119 (from eBioscience). For HSC sorting, the debris, dead and clumped cells were firstly removed to obtain the single and viable cells, then the Sca-1 positive, c-Kit positive and Lineage negative cell population were isolated by HSC sorting, and the FACS analysis was performed on BD FACSMelody™ Cell Sorter (2).

**Isolation of mouse PBMC cells**. Heparinized peripheral blood was collected from mouse subjects by heart puncture and diluted 1:3 with Hank's balanced salts solution without Ca^2+^/Mg^2+^ (HBSS solution). The diluted blood was layered onto 10 ml of Ficoll-Paque in 15 ml sterile centrifuge tubes followed by centrifugation at 300×g at 20ºC for 40 min. The PBMC layers were then harvested and washed by HBSS solution. The pellets were resuspended with lysing buffer containing 150 mM NH4Cl, 1.0 mM KHCO3, and 0.1 mM Na2EDTA, pH 7.4 and incubated for 5 min at room temperature to remove contaminated red cells. The cell suspensions were then centrifuged and washed with HBSS solution before the cell pellet was resuspended for further biomedical analysis.

**Isolation of mouse intestine epithelial cells (IEC).** The protocol for isolation of IEC cells was based on the previously described method with minor modifications. In brief, the small and large intestines were harvested individually from treated mice and rinsed extensively with RPMI-1640 media (from Lonza) after Peyer’s patches were removed (for small intestine). The rinsed intestines were opened longitudinally and macerated; the tissue pieces were shaken gently in RPMI-1640 containing 2 mM EDTA and 10% fetal calf serum. The tissue preparations were passed through 70-μm mesh filters, and the resulting single-cell suspensions were applied to Percoll (from Sigma) density gradients of 25%, 40%, and 75%. After centrifugation at 2,000×g for 20 min, the interface between the 25% and 40% layers was collected to obtain IECs. The cells were stained using antibodies for either epithelial cell adhesion molecule (EpCAM, from Biolegend) or CD45 (from Biolegend) and nucleic acid dye (Via-Probe, from BD Biosiences). The Via-Probe^-^/CD45^-^/EpCAM^+^ IEC were sorted using BD FACSMelody^TM^ Cell Sorter (BD Biosciences) for further biomedical analysis (16, 17).

**Intestinal permeability assay.** The protocol was followed based on the previously described method with minor modifications. In brief, treated mice were fasted for 4 h before the experiment and then the FITC-dextran (50mg/mL, Cat# 46944 from Sigma) was gavaged to mice (600 mg/kg). After 4 h, the whole blood was collected by cardiac puncture and placed at room temperature for 1 h before being centrifuged at a speed of 3000 rpm for 10 min. The supernatant was then transferred to a new tube for further centrifugation at a speed of 12,000 rpm for 10 min at 4 °C. The subsequent supernatant (serum) was diluted with equal volume of PBS and 100μL diluted serum was added to a 96-cell microplate. The concentration of FITC in serum was determined at excitation/emission wavelengths of 485/530nm using a FLx800 microplate fluorescence reader (Bio-Tek). The serial diluted FITC-dextran (0, 0.5, 1, 2, 4, 6, 8, 10 μg/μL) was used as standards. Serum of mice administered with PBS was used as negative controls (18, 19).

**Fecal microbiome analysis.** Fecal samples of the experimental mice were collected and stored at −80°C before being processed. Microbial DNA was extracted using a QIAamp Fast DNA Stool Mini Kit (from Qiagen) according to the manufacturer's protocol (20). The purity and concentration of the extracted DNA were detected using agarose gel electrophoresis. Fecal microbiota were studied by performing V3-V4 16S rDNA amplicon sequencing in order to obtain the operational taxonomic units (OTU) defining the bacterial communities (21). Sequencing samples from frozen fecal pellets were prepared, sequenced and subsequently processed using the MiSeq Pe300 Sequencing Platform (from Illumina) by Shanghai OE Biotech Inc. The raw data were treated and processed using a QIIME 2^TM^ software package, and the subsequent sequences of OTU were blasted in the Silva database (version 138). The alpha diversity and beta diversity were analyzed using QIIME 2^TM^ software package (18).

**Serum biochemical analysis**. The whole blood was collected from experimental mice by heart puncture and the serum was prepared by centrifugation at 2000g for 15 min. The specimens were then stored at −80°C until analysis. The GSH/GSSG ratio was determined using a GSH/GSSG Ratio Detection Assay Kit (Fluorometric - Green) (#ab138881) from Abcam, while diamine oxidase (DAO) activity was measured by DAO ELISA Kit (#[MBS160374](https://www.mybiosource.com/mouse-elisa-kits/diamine-oxidase-dao/160374)) and the zonulin levels were determined by Mouse Zonulin ELISA Kit (#NC1314884) from MyBioSource according to manufacturers’ instructions (22, 23).

**Analysis of cytokines.** Mouse cytokines from either the serum or cell supernatant, including IL1β, IL6, IL17a and MCP1 were measured using the Bio-Plex Pro Mouse Cytokine 23-plex Assay kit (#M60009RDPD from BioRad) and Bio-Plex 200 Systems (BioRad) according to manufacturer’s instructions. Protein concentration in lysates was determined using the Coomassie Protein Assay Kit (Pierce Biotechnology) according to the manufacturer’s instructions and lysates were adjusted to 200 mg/ml with extraction buffer. 50 mL lysate were diluted 2× in sample dilution buffer and analyzed in duplicates. Analytes were quantified in each sample against a calibration curve of known concentrations (24).

**Immunostaining**. The treated cells were transferred to cover slips and the cells were fixed in 4% paraformaldehyde for 20 min before being incubated with 0.3% Triton X-100 in PBS for 15 min. After blocking with 5% goat serum in PBS at room temperature for 30 min, cells were incubated with antibody for either CLDN1 or 8-oxo-dG (# 4354-MC-050, from Novus Biologicals) for 12 h at 4°C and subsequently with secondary antibody Alexa Fluor 488. The cover slips were then mounted by antifade Mountant with DAPI (staining nuclei, in blue). The photographs were taken using a Confocal Laser Microscope (Leica, 20x lens) and quantitated by Image J. software (1).

**Statistical analysis**. The data was given as mean ± SEM and all the experiments were performed at least in quadruplicate unless otherwise indicated. The one-way analysis of variance (ANOVA) followed by the Turkey−Kramer test was used to determine statistical significance of different groups by SPSS 22 software, and a *P* value of <0.05 was considered significant.

**References**

1. Wang X, Lu J, Xie W, Lu X, Liang Y, Li M, et al. Maternal diabetes induces autism-like behavior by hyperglycemia-mediated persistent oxidative stress and suppression of superoxide dismutase 2. *Proc Natl Acad Sci U S A.* 2019;116(47):23743-52.

2. Xie W, Zhou X, Hu W, Chu Z, Ruan Q, Zhang H, et al. Pterostilbene accelerates wound healing by modulating diabetes-induced estrogen receptor beta suppression in hematopoietic stem cells. *Burns Trauma.* 2021;9:tkaa045.

3. Ceradini DJ, Yao D, Grogan RH, Callaghan MJ, Edelstein D, Brownlee M, et al. Decreasing intracellular superoxide corrects defective ischemia-induced new vessel formation in diabetic mice. *J Biol Chem.* 2008;283(16):10930-8.

4. Zhang H, Li L, Li M, Huang X, Xie W, Xiang W, et al. Combination of betulinic acid and chidamide inhibits acute myeloid leukemia by suppression of the HIF1alpha pathway and generation of reactive oxygen species. *Oncotarget.* 2017;8(55):94743-58.

5. Yao D, Shi W, Gou Y, Zhou X, Yee Aw T, Zhou Y, et al. Fatty acid-mediated intracellular iron translocation: a synergistic mechanism of oxidative injury. *Free Radic Biol Med.* 2005;39(10):1385-98.

6. Eads CA, Danenberg KD, Kawakami K, Saltz LB, Blake C, Shibata D, et al. MethyLight: a high-throughput assay to measure DNA methylation. *Nucleic Acids Res.* 2000;28(8):E32.

7. Nosho K, Irahara N, Shima K, Kure S, Kirkner GJ, Schernhammer ES, et al. Comprehensive biostatistical analysis of CpG island methylator phenotype in colorectal cancer using a large population-based sample. *PLoS ONE.* 2008;3(11):e3698.

8. Xie W, Ren M, Li L, Zhu Y, Chu Z, Zhu Z, et al. Perinatal testosterone exposure potentiates vascular dysfunction by ERbeta suppression in endothelial progenitor cells. *PLoS One.* 2017;12(8):e0182945.

9. Silverman JL, Yang M, Lord C, and Crawley JN. Behavioural phenotyping assays for mouse models of autism. *Nat Rev Neurosci.* 2010;11(7):490-502.

10. Schaafsma SM, Gagnidze K, Reyes A, Norstedt N, Mansson K, Francis K, et al. Sex-specific gene-environment interactions underlying ASD-like behaviors. *Proc Natl Acad Sci U S A.* 2017;114(6):1383-8.

11. Moy SS, Nadler JJ, Perez A, Barbaro RP, Johns JM, Magnuson TR, et al. Sociability and preference for social novelty in five inbred strains: an approach to assess autistic-like behavior in mice. *Genes Brain Behav.* 2004;3(5):287-302.

12. Xie W, Ge X, Li L, Yao A, Wang X, Li M, et al. Resveratrol ameliorates prenatal progestin exposure-induced autism-like behavior through ERβ activation. *Mol Autism.* 2018;9:43.

13. Yao D, Vlessidis AG, Gou Y, Zhou X, Zhou Y, and Evmiridis NP. Chemiluminescence detection of superoxide anion release and superoxide dismutase activity: modulation effect of Pulsatilla chinensis. *Anal Bioanal Chem.* 2004;379(1):171-7.

14. Kong D, Zhan Y, Liu Z, Ding T, Li M, Yu H, et al. SIRT1-mediated ERbeta suppression in the endothelium contributes to vascular aging. *Aging Cell.* 2016.

15. Rossi L, Challen GA, Sirin O, Lin KK, and Goodell MA. Hematopoietic stem cell characterization and isolation. *Methods Mol Biol.* 2011;750:47-59.

16. Lee J, Mohsen A, Banerjee A, McCullough LD, Mizuguchi K, Shimaoka M, et al. Distinct Age-Specific miRegulome Profiling of Isolated Small and Large Intestinal Epithelial Cells in Mice. *Int J Mol Sci.* 2021;22(7).

17. Lee J, Park EJ, Yuki Y, Ahmad S, Mizuguchi K, Ishii KJ, et al. Profiles of microRNA networks in intestinal epithelial cells in a mouse model of colitis. *Sci Rep.* 2015;5:18174.

18. Li Y, Luo ZY, Hu YY, Bi YW, Yang JM, Zou WJ, et al. The gut microbiota regulates autism-like behavior by mediating vitamin B6 homeostasis in EphB6-deficient mice. *Microbiome.* 2020;8(1):120.

19. Hsiao EY, McBride SW, Hsien S, Sharon G, Hyde ER, McCue T, et al. Microbiota modulate behavioral and physiological abnormalities associated with neurodevelopmental disorders. *Cell.* 2013;155(7):1451-63.

20. Tabouy L, Getselter D, Ziv O, Karpuj M, Tabouy T, Lukic I, et al. Dysbiosis of microbiome and probiotic treatment in a genetic model of autism spectrum disorders. *Brain Behav Immun.* 2018;73:310-9.

21. Cristiano C, Pirozzi C, Coretti L, Cavaliere G, Lama A, Russo R, et al. Palmitoylethanolamide counteracts autistic-like behaviours in BTBR T+tf/J mice: Contribution of central and peripheral mechanisms. *Brain Behav Immun.* 2018;74:166-75.

22. Esnafoglu E, Cirrik S, Ayyildiz SN, Erdil A, Erturk EY, Dagli A, et al. Increased Serum Zonulin Levels as an Intestinal Permeability Marker in Autistic Subjects. *J Pediatr.* 2017;188:240-4.

23. Xu Y, Xie L, Zhang Z, Zhang W, Tang J, He X, et al. Tremella fuciformis Polysaccharides Inhibited Colonic Inflammation in Dextran Sulfate Sodium-Treated Mice via Foxp3+ T Cells, Gut Microbiota, and Bacterial Metabolites. *Front Immunol.* 2021;12:648162.

24. Sharon G, Cruz NJ, Kang DW, Gandal MJ, Wang B, Kim YM, et al. Human Gut Microbiota from Autism Spectrum Disorder Promote Behavioral Symptoms in Mice. *Cell.* 2019;177(6):1600-18 e17.
